# Mechanistic modulation of electronic structure and interface chemistry in Ni-based alloys for electrochemical energy conversion

**DOI:** 10.1039/d5ra09308b

**Published:** 2026-04-21

**Authors:** Akrajas Ali Umar, Munetaka Oyama

**Affiliations:** a Institute of Microengineering and Nanoelectronics, Universiti Kebangsaan Malaysia 43600 UKM Bangi Selangor Malaysia akrajas@ukm.edu.my; b Nanomaterials Chemistry Laboratory, Department of Materials Chemistry, Graduate School of Engineering, Kyoto University Nishikyo-ku 816-6520 Kyoto Japan

## Abstract

Nickel (Ni) alloys are central to next generation electrocatalysts for clean energy conversion, driving key reactions such as the hydrogen evolution reaction (HER), oxygen evolution reaction (OER), and electrochemical CO_2_ reduction. Despite extensive performance reports, the mechanistic role of alloying, how it reshapes Ni's structure, electronic states, and surface chemistry to boost activity, remains insufficiently understood. This review moves beyond application-driven summaries to dissect the physicochemical transformations induced by alloying, through d-band modulation, orbital hybridization, and interfacial charge redistribution, and their direct impact on catalytic function. We integrate insights across structural, electronic, and interfacial scales, linking compositional engineering to performance metrics. State-of-the-art synthesis strategies are evaluated, and emerging design principles for durable, high-efficiency Ni-based catalysts are outlined. By uniting mechanistic understanding with material innovation, this work provides a roadmap for accelerating the development of robust, sustainable electrochemical energy systems.

## Abstract 2

Alloying in Ni catalyst modifies electronic, surface chemistry and surface structure properties, generating novel catalytic properties.

## Graphical abstract

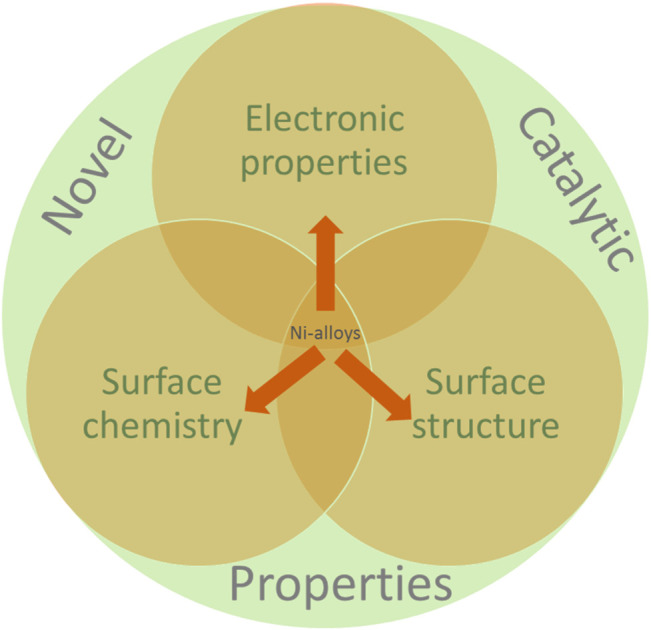

## 1. Introduction

The pursuit of sustainable and efficient energy solutions has become increasingly critical in the face of rising energy demands. Among the various technologies being explored, electrochemical systems, particularly those utilizing alloyed nickel (Ni) electrodes, have emerged as promising candidates for advancing clean energy applications.^1–4^ Ni, a transition metal known for its cost-effectiveness and favorable catalytic properties, serves as a versatile substrate in these systems. Ni's unique properties make it a valuable catalyst in various processes, particularly in hydrogenation reactions where it converts unsaturated fats to saturated fats and selectively hydrogenates alkynes to alkenes, such as transforming acetylene into ethylene.^5,6^ It also effectively adsorbs hydrogen (H_2_) and reactant molecules on its surface, facilitating the addition of hydrogen atoms to produce reduced products.^7^ In methanation and CO_2_ conversion, Ni catalyzes the Sabatier reaction, combining CO_2_ with hydrogen to generate methane and water, which is essential for synthetic natural gas production and addressing carbon emissions.^8–11^ Its cost-effectiveness and high activity also make Ni a preferred catalyst in steam methane reforming (SMR), where it efficiently activates methane to produce hydrogen and carbon monoxide without excessive carbon build-up.^12–14^ Furthermore, in electrocatalysis, Ni is utilized in hydrogen evolution reactions (HER) during alkaline electrolysis to generate hydrogen gas from water^15–17^ and oxygen evolution reaction (OER) to produce oxygen from water.^15,18,19^

Despite these advantages, Ni suffers from moderate intrinsic activity, limited stability, and susceptibility to corrosion, which restrict its performance under the demanding conditions of practical energy applications. To overcome these limitations, alloying and alloying strategies have been widely adopted.^20,21^ Incorporating metals such as platinum (Pt), palladium (Pd), gold (Au), or rhodium (Rh) into Ni frameworks^22,23^ generates synergistic effects that optimize electronic structures, tune adsorption energies, and increase the density of active sites. These modifications not only enhance catalytic activity and durability but also mitigate issues such as carbon deposition and surface passivation. Alloyed Ni systems thus represent a critical pathway toward achieving the balance of performance, cost, and sustainability required for next-generation energy technologies. By incorporating these metals, researchers have observed substantial enhancements in catalytic activity, stability, and overall efficiency in key electrochemical reactions, including HER, OER, and various organic transformations. The synergistic effects arising from the interactions between Ni and metals lead to optimized electronic structures and improved active site availability, which are crucial for enhancing reaction kinetics.

This review provides a mechanistic and design-oriented perspective on Ni-based alloy catalysts for sustainable energy applications. We highlight how alloying alters the electronic configuration, geometric structure, and surface chemistry of Ni, thereby advancing its catalytic properties. Particular attention is given to mechanistic insights into their application in electrochemical, such as HER, OER, CO_2_ reduction, and hydrocarbon reforming, where Ni alloys demonstrate unique reactivity patterns. Their performance in thermochemical applications, such as Sabatier reaction, hydrogenation, are also discussed to further emphasize how alloying affects their performance in this process. In addition, we discuss design strategies and modification techniques—including physical deposition, chemical vapor deposition, and galvanic replacement—that enable precise control over catalyst architecture. By synthesizing current knowledge and identifying gaps in mechanistic understanding, this review aims to establish guiding principles for the rational design of Ni-based alloys. Ultimately, we seek to chart a roadmap for innovative catalyst engineering that will accelerate the deployment of sustainable energy solutions and contribute to the global transition toward a low-carbon future.

## 2. Origin of catalytic activity in Ni

Ni is a d-band metal, containing 28 electrons with an electron configuration of [Ar] 3d^8^ 4s^2^. The properties of 3d and 4s outer shell with 10 electrons contribute to the unique properties of Ni.^24,25^ As a transition metal, Ni exhibits a propensity to form stable oxidation states, most notably +2 and +3, by the loss of its 4s and, subsequently, 3d electrons.^26–28^ On this basis, Ni has partially filled d-orbital. The presence of partially filled d-orbitals plays a crucial role in the metal's chemical reactivity and bonding capabilities with reactant molecules.^29^ These d-orbitals also facilitate effective adsorption and activation of various molecules, including hydrogen (H_2_), carbon monoxide (CO), and oxygen (O_2_).^30^ Furthermore, electrons in d orbital allows Ni to engage in various coordination modes with ligands, which are ions or neutral molecules that can donate electron pairs to form coordinate covalent bonds. Ni crystallizes in a face-centered cubic (fcc) structure at room temperature.^31^ The fcc lattice is notable for its high packing efficiency, allowing for a maximum atomic density of approximately 74%. In this lattice configuration, the atoms occupy the corners and the centers of the cube faces, resulting in exceptional ductility and malleability. At room temperature, Ni has a lattice parameter of approximately 3.52 Å, which remains stable up to around 1450 °C, beyond which it begins to undergo phase transitions that alter its properties.

In addition to its fcc structure, nickel can also adopt a hexagonal close-packed (hcp) configuration under high pressures and temperatures, a behavior crucial for its performance in extreme environments such as aerospace engineering.^32^ The mechanical properties of nickel are closely tied to its atomic structure and the presence of dislocations within its crystal lattice, contributing to its high tensile strength of about 370 MPa in the annealed state and significant ductility, allowing it to deform without fracturing—an advantageous trait for applications requiring complex shaping. Furthermore, the presence of nickel in austenitic stainless steels, for instance, significantly improves their resistance to pitting and crevice corrosion, making them suitable for applications in harsh environments, including marine and chemical processing. Nickel exhibits a coefficient of thermal expansion of approximately 13.4 × 10^−6^ °C^−1^, which indicates that it expands and contracts moderately with temperature fluctuations. This property is essential to consider in applications involving thermal cycling, such as in cryogenic systems or environments with substantial temperature variations, where incompatible thermal expansion coefficients can lead to stresses and potential failure. Moreover, Ni's catalytic properties are exploited in the production of hydrogen and as a catalyst in various chemical reactions. The structural stability, combined with the fcc lattice arrangement, allows nickel to function effectively in these roles, facilitating surface reactions that are critical for industrial processes. Additionally, advancements in battery technology have prompted increased interest in nickel-rich cathode materials for lithium-ion batteries, where its specific structural properties contribute to improved energy density and stability.

While nickel catalysts provide a compelling case for their use in various catalytic applications, their challenges, notably deactivation through poisoning and structural instability, cannot be overlooked. The tendency for these catalysts to succumb to external impurities and structural changes impedes their long-term effectiveness and efficiency in industrial processes. The most critical hurdles to the effective utilization of Ni catalysts is catalyst poisoning. Poisoning occurs when foreign substances, which are often present in the reactants or environment, adsorb onto the catalyst surface, inhibiting its active sites and ultimately leading to reduced catalytic activity. Various species, including sulfur compounds, carbon monoxide (CO), and even halides, are known to be particularly detrimental to nickel catalysts.^33^ For example, sulfur compounds, the presence of sulfur can readily form nickel sulfide on Ni surface, which is often inactive in catalytic processes. This reaction not only reduces the available active sites but also alters the electronic properties of the nickel, further impairing its ability to facilitate desired reactions. In carbon monoxide poisoning, especially, two distinct mechanisms must be differentiated. In thermochemical catalysis, CO can undergo disproportionation or transformation into solid carbon deposits (coking), leading to irreversible deactivation of Ni surfaces.^34,35^ In contrast, in electrochemical environments, the more relevant phenomenon is CO adsorption (CO_ads_) poisoning, where CO molecules adsorb strongly onto Ni active sites, blocking hydrogen adsorption and hindering reaction turnover.^36,37^ This competitive adsorption reduces hydrogenation activity and severely impacts the hydrogen evolution reaction (HER) and oxygen evolution reaction (OER). Even trace levels of CO_ads_ have been shown to significantly suppress Ni's catalytic efficiency, as the strong Ni–CO bond stabilizes surface complexes and prevents access to active sites. For example, in alkaline HER, CO_ads_ competes directly with H adsorption, lowering reaction kinetics without necessarily transforming into solid carbon. This reversible but persistent poisoning effect is a major challenge in electrochemical CO_2_ reduction and hydrocarbon reforming, where CO intermediates are abundant. Therefore, it is essential to distinguish between irreversible coking and reversible CO_ads_ poisoning when discussing Ni catalyst deactivation. Alloying strategies—such as incorporating Pt, Au, or Bi—have been reported to mitigate CO_ads_ poisoning by tuning adsorption energies and facilitating faster desorption of CO, thereby restoring catalytic activity.^38–40^

In addition to poisoning, structural instability remains a pressing challenge associated with nickel catalysts.^41^ Structural stability, which pertains to the catalyst's ability to maintain its geometric and electronic configuration during the catalytic process, is crucial for ensuring prolonged catalytic activity. Ni catalysts can undergo morphological changes, sintering, or leaching, especially under harsh catalytic conditions, thereby impairing their performance.^42,43^ Sintering, for example, involves the agglomeration of catalyst particles, leading to a decrease in the surface area available for reaction. In nickel catalysts, this phenomenon can occur at elevated temperatures and can significantly alter the catalyst structure, diminishing its activity. For instance, as Ni particles sinter, there is a marked reduction in the number of available active sites, resulting in an overall decrease in catalytic efficiency. Moreover, larger Ni particles tend to exhibit less favorable catalytic behavior compared to their smaller counterparts due to the difference in electronic properties and accessibility of active sites.^41^ For the case of leaching, the structural instability that can severely compromise the effective use of nickel catalysts, it occurs when Ni ions dissolve into the reaction medium, particularly under acidic or corrosive conditions.^44,45^ This dissolution not only leads to a loss of the active catalyst phase but also can potentially cause environmental and economic issues, particularly if the catalyst is designed to be reused. Moreover, the leaching of nickel can have adverse effects on the selectivity of certain reactions, often leading to unwanted by-products.

However, the available drawback in Ni have been continuously overcome. For example, Ni's catalytic and structural performance can be significantly enhanced through alloying with other metals, such as platinum (Pt), palladium (Pd), and copper (Cu). These alloyed systems often exhibit improved stability and activity compared to pure nickel catalysts. The development of nanoporous nickel structures and nickel-based core–shell catalysts has further advanced the field, as these configurations increase the density of active sites and improve overall reactivity. Recent research has demonstrated that engineering the morphology of nickel catalysts can lead to substantial enhancements in catalytic performance, particularly in reactions involving complex substrates.

## 3. Electronic state modulation by alloying

When Ni is alloyed with metals, one of the primary interaction effects is charge transfer between the metals.^46,47^ This charge transfer facilitates electron redistribution at their interface, which tunes the electronic properties of Ni. As a result, the electronic density of states is modified, leading to a shift in the d-band center.^48^ This electronic structure tuning process in Ni-alloy systems, for instance, also involves modifying the electronic density of states at the surface to optimize catalytic behavior.^49^

**Fig. 1:**
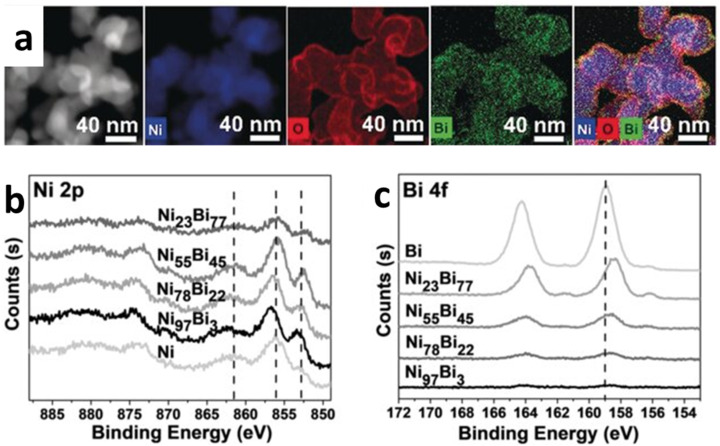
Electronic structure tuning in alloyed Ni. (a) Elemental mapping of Ni_97_Bi_3_ bimetallic nanostructure. (b and c) XPS spectra of the bimetallic nanostructure with different concentration of Bi alloying. Binding energy of Ni 2p and Bi 4f are shifted, modifying the d-band center of the Ni catalyst (adapted from ref. 40, © 2020 Wiley-VCH Verlag GmbH & Co. KGaA, Weinheim).

The d-band center is a critical parameter affecting the adsorption strength of reactants on the catalyst surface. A d-band centre closer to the Fermi level generally increases adsorption strength beneficial for activating reactants but may result in stronger binding of intermediates, making product release harder. Conversely, a d-band center farther from the Fermi level weakens adsorption, enhancing product release but potentially reducing activation efficiency. A recent study demonstrates this process.^40^ By alloying Ni with Bi to form a bimetallic nanostructure Ni_97_Bi_3_, an optimum shift of the d-band center of Ni was obtained. In this study, the successful formation of the NiBi bimetallic system is obtained from the EDS elemental mapping. Meanwhile, the tuning of the electronics structure of the Ni by the alloying is indicated by the shifting in the Ni (2p) binding energy along with the shifting in the Bi (4f) binding energy as obtained from the XPS spectroscopy result. The results are shown in Fig. 1. This alloying also causes large compression strain in the Ni lattice and greatly improve the conductivity of the bimetallic system. The electrocatalysis using this Ni_97_Bi_3_ bimetallic nanostructure exhibit excellent performance in methanol oxidation. It was reported that the Bi alloying into the Ni lattice create an abundant active site for catalytic reaction, improve electron and mass transfer during the reaction and resist the CO poisoning.

Alloying Ni with noble metals such as platinum (Pt) or palladium (Pd) creates electronic synergy, where the electronic structures of the metals interact to optimize catalytic properties. For instance, in Pt–Ni alloys, the negatively charged Pt (Pt^*δ*−^) and positively charged Ni (Ni^*δ*+^) facilitate favorable interactions with reactants, leading to enhanced catalytic activity.^50^ Furthermore, alloying Ni with noble metals like Pt leads to strong electronic hybridization between the d orbitals of the metals. The 5d orbital of Pt and the 3d orbital of Ni interact, which modulates the d band center of Pt. This modification boosts the hydrogen evolution reaction (HER) activity and stabilizes the Pt single atoms on the Ni surface.^51^ Similar phenomenon was also observed when Ni was alloyed with metal, like Ti, Ge and Sn, where the electronic structure changes influence the bonding scheme and adsorption energy of hydrogen atoms on the active sites, which is crucial for HER efficiency.^52^

Phenomenon of electronics structure adjustment in Ni upon alloying that enhance the catalytic performance of the alloy system can be seen in the NiFe alloys system.^53^ It is discovered that effective modulation in the local electronic structure of Ni–Fe layered double hydroxide (LDH) is occurred by strong interfacial interactions with FeOOH nanoparticles (NPs). This FeOOH/LDH composites system exhibits excellent performance in OER catalysis application with low overpotential. This performance is comparable to the noble metal OER catalyst system.^54^ The local electronics structure modification was occurred in the new system the result of the presence of high-oxidation-state Fe^(3+*δ*)+^ sites with relatively short Fe^(3+*δ*)+^–O bond from the highly unsaturated ultrafine FeOOH NPs. Thus, electronics oxidation and electrocatalytic behavior of the alloyed Ni based composite system become magnified. In the system of Ni–Fe alloy embedded in the N-doped carbon nanobox, similar catalytic OER properties enhancement was also observed. Analytical study using ultraviolet photoelectron spectroscopy and theoretical simulation found that the alloying of Ni into Fe modulates the electronic structure, favoring intermetallic charge-transfer and downshifting the d-band center of Fe adsorption sites. This effectively lowers the reaction barriers of the electrocatalytic oxygen reduction reaction (ORR)/OER processes.^55^ In the typical measurement, the catalytic performance of the Ni based composite electrode outperformed the commercialized noble-metal system with a half-wave potential of 0.891 V for ORR and a small overpotential of 325 mV at 10 mA cm^−2^ for OER both in 0.1 M KOH solution. Similar mechanism is also observed in Ni-MOF-system co-alloyed with Fe and Se where the alloying process fine-tuned the electronics structure, enhancing the surface chemistry properties of the catalyst. This phenomenon then optimized the OER catalytic activity with low overpotential of 242 mV.^56^

**Fig. 2:**
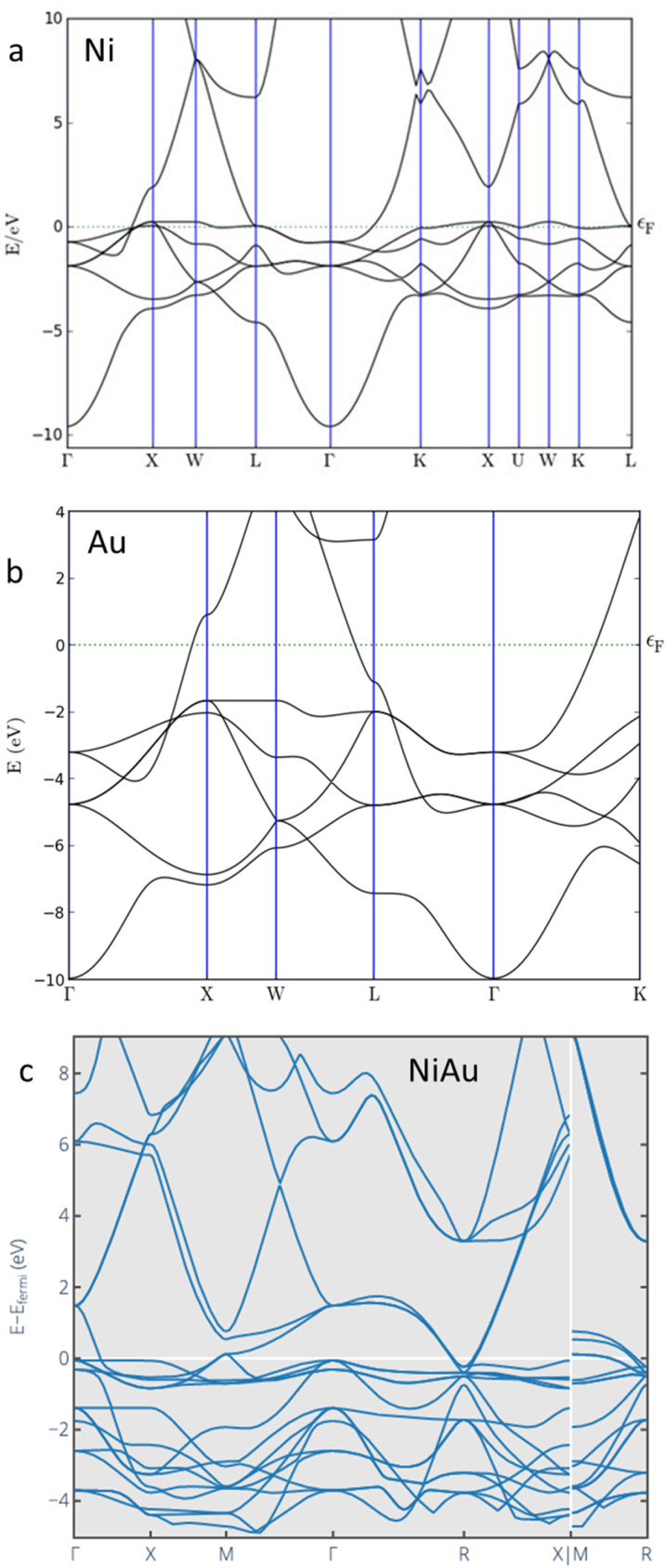
Band structures of (a) Ni, (b) Au, and (c) NiAu alloy. Pure Ni and Au adopt a face-centered cubic (fcc) structure with space group *Fm*3̄*m*, whereas NiAu crystallizes in an orthorhombic uranium silicide-type structure with space group *Pnma* (adapted from ref. 62, © 2020 http://Osti.gov).

In pure Ni, the band structure exhibits a distinct distribution of energy bands characterized by a filled d band and a range of unfilled states above the Fermi level.^57^ The introduction of metals, for example Au, into Ni system leads to hybridization effects in its electronic energy system where the d-levels of Ni interact with the s and d-bands of Au. This can alter the band structure of Ni, leading to either band filling or band widening, which affects the overall electronic properties. Fig. 2 shows the modification of Ni band structure when being alloyed with Au. While Ni and Au individually adopt fcc cubic structures (space group *Fm*3̄*m*), NiAu alloys crystallize in an orthorhombic uranium silicide-type structure (space group *Pnma*), which significantly alters the electronic band structure compared to the parent metals. This structural transformation modifies the d-band center of Ni, thereby tuning adsorption energies and lowering reaction barriers. Such alloying has been shown to enhance catalytic properties, particularly in electrochemical proton reduction and CO_2_ reduction, as supported by recent experimental and theoretical studies.^58–61^

**Fig. 3:**
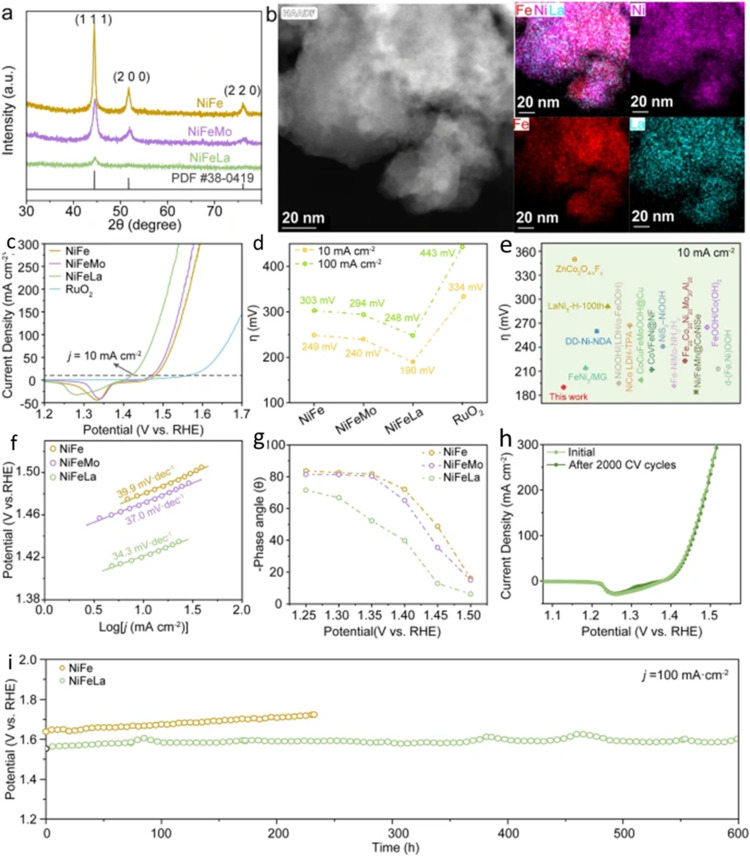
Electronic properties of NiFe, NiFeMo and NiFeLa. (a) Corresponding XRD patterns for NiFe, NiFeMo and NiFeLa. (b) HAADF-STEM image along with elemental mappings of NiFeLa. (c) The Linear Sweep Voltammetry (LSV) curves of the samples in 1 M KOH solution and commercial RuO_2_ under oxygen-saturated conditions. (d) Comparison of overpotential of the samples at current densities of 10 and 100 mA cm^−2^. (e) The overpotential of NiFeLa at 10 mA cm^−2^ compared with transition metal-based Oxygen Evolution Reaction (OER) electrocatalysts. (f) The corresponding Tafel plots of the samples. (g) The phase peak angles of the materials within a specific voltage range. (h) The typical CV stability performance of NiFeLa in 1 M KOH solution. (i) Stability properties of the NiFeLa catalyst *via* LSV curves before and after 2000 cyclic voltammetry cycles (adapted from ref. 18, © 2024 Springer Nature).

The following results present recent findings on how alloying in Ni-based catalysts enhances catalytic performance through d-orbital hybridization (Fig. 3).^18^ The modification of the d-orbital in Ni based catalyst (NiFe) is observed when a third metal (La or Mo) is introduced. As presented in the figure, the La alloying optimized hybridization between d orbital in NiFeLa and 2p in oxygen, enabling enhanced adsorption strength of oxygen intermediates, and reduced rate-determining step energy barrier, parameters that is crucial for the enhanced OER performance. The ternary NiFeLa catalyst demonstrates outstanding performance, reaching a current density of 1 A cm^−2^ at a remarkably low operating potential of 1.58 V in an anion exchange membrane electrolyzer. Furthermore, it exhibits robust long-term stability, maintaining performance for at least 600 h.

**Fig. 4:**
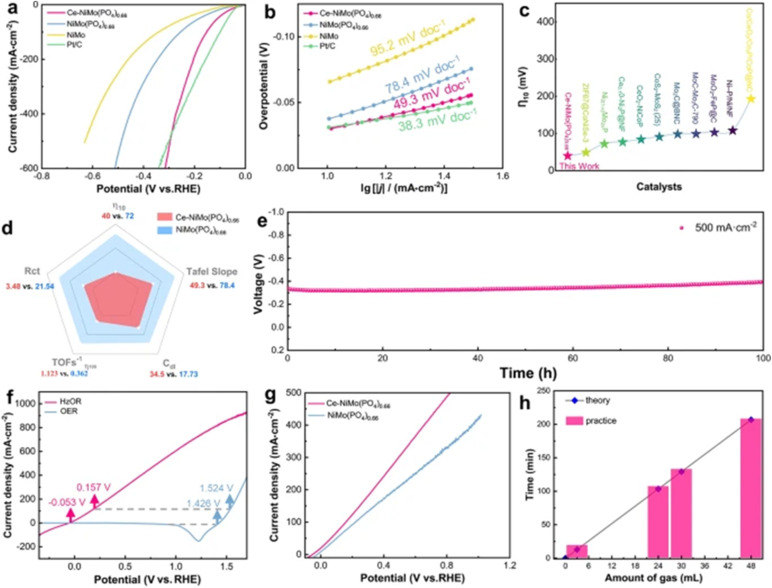
(a) Polarization curves of Ce-NiMo(PO_4_)_0.66_, NiMo(PO_4_)_0.66_, NiMo, and Pt/C in 1 M KOH; (b) a comparative analysis of corresponding Tafel slopes; (c) a performance benchmark of Ce-NiMo(PO_4_)_0.66_ against recent findings in the field; (d) an integrated electrochemical performance radar chart; (e) chronopotentiometric measurements of Ce-NiMo(PO_4_)_0.66_ at a constant current density of 500 mA cm^−2^ in 1 M KOH electrolyte; (f) a comparison of linear sweep voltammetry (LSV) polarization curves of Ce-NiMo(PO_4_)_0.66_ for both hydrazine oxidation reaction (HzOR)-assisted and non-assisted hydrogen evolution reaction (HER) and oxygen evolution reaction (OER); (g) polarization curves of Ce-NiMo(PO_4_)_0.66_ and NiMo(PO_4_)_0.66_ in 1 M KOH supplemented with 0.5 M N_2_H_4_; and (h) the determination of the Faraday efficiency of H_2_ production using Ce-NiMo(PO_4_)_0.66_ (adapted from ref. 2, © 2024 Springer Nature).

Fig. 4 presents an additional noteworthy finding, demonstrating how orbital hybridization in the Ni-based alloy (Ce-NiMo(PO_4_)_0_._66_) enhances catalytic performance in the HER process.^2^ Ce-NiMo(PO_4_)_0.66_ exhibits remarkable alkaline HER performance, achieving overpotentials of only 40 mV and 295 mV at current densities of 10 mA cm^−2^ and 500 mA cm^−2^, respectively, and demonstrates robust long-term durability at an industrial current density of 500 mA cm^−2^. Furthermore, an overall hydrazine splitting (OHzS) system employing Ce-NiMo(PO_4_)_0.66_ as a bifunctional electrocatalyst for both hydrazine oxidation reaction (HzOR) and HER achieves industrial current densities at a low voltage of 0.92 V, confirming its potential for sustainable hydrogen production and hydrazine pollutant degradation. It has been obtained that the incorporation of Ce, with its characteristic enriched 4f electronic distribution near the Fermi level, modulates the 3d–2p orbital hybridization. This modulation optimizes the electronic structure of the nickel–molybdenum phosphate, synergistically enhancing water dissociation and proton dehydrogenation transfer within the hydrogen evolution reaction (HER) through the cooperative action of nickel–molybdenum dual sites and the phosphate active unit. This study further highlights the effectiveness of manipulating 3d–2p hybridization *via* the introduction of 4f orbital electronic states as a strategy for improving the HER activity of transition metal compound catalysts.

**Fig. 5:**
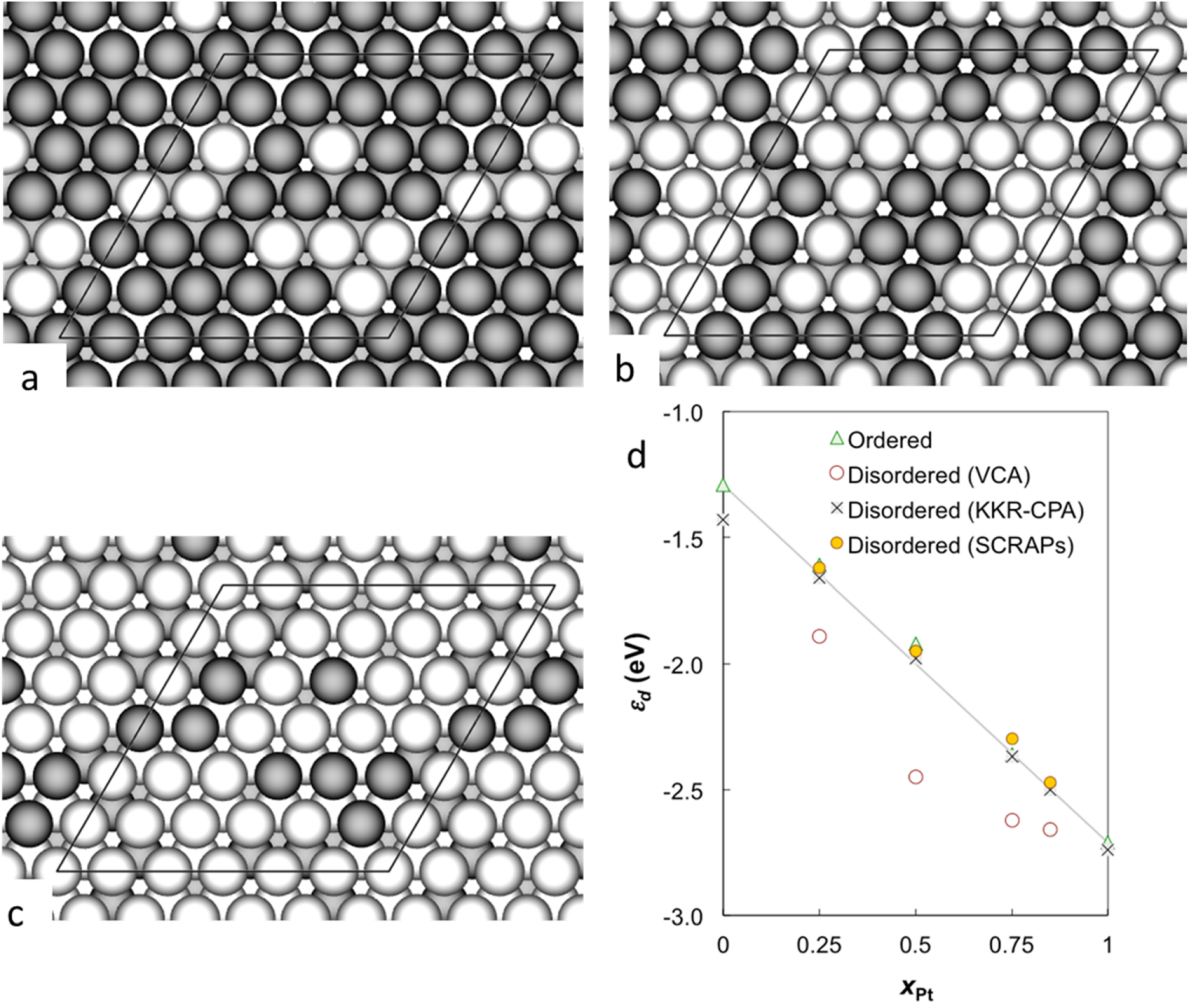
The surface geometries from top of (6 × 6) (111) alloy surfaces of Pt and Ni with varying compositions – (a) Pt_0_._25_Ni_0_._75_, (b) Pt_0_._5_Ni_0_._5_, and (c) Pt_0_._75_Ni_0_._25_. Pt and Ni atoms are distinguished by lighter and darker spheres, respectively, with surface unit cells delineated. (d) The d-band center (*ε*_d_) for both monometallic Pt and Ni, and for the PtNi alloys, is presented as a function of Pt atomic composition (*x*Pt). These calculations are derived from Virtual Crystal Approximation (VCA), Korringa–Kohn–Rostoker Coherent Potential Approximation (KKR-CPA), and Super-Cell Random Approximates (SCRAPs) methodologies (adapted from ref. 65, © 2020 Springer Nature).

The charge density redistribution at the interface of Ni–Pt alloy surfaces can be estimated by using DFT modelling (Fig. 5). The charge transfer between Ni and Pt atoms in the Ni–Pt alloys is normally studied by the core-level shifts of Ni 2p_3/2_ and Pt 4f_7/2_ X-ray photoelectron spectra. Charge density analysis show that the electron transfer occurred from Ni to Pt, attributed to the higher electronegativity of Pt. This redistribution reduces (Fig. 5a–c) the charge density on Pt's surface atoms, lowering hydrogen adsorption energy and improving desorption kinetics.^63,64^ Simulations reveal that charge transfer increases with Ni content up to a threshold (*e.g.*, a 50 : 50 alloy), beyond which excessive Ni dilutes Pt's activity. Calculation of the adsorption energy of hydrogen (Δ*E*_H*_) (Fig. 5d), a critical parameter that quantifies the energy required to adsorb or release a hydrogen atom on the catalyst surface, over the catalyst found that for HER, the ideal Δ*E*_H*_ is close to 0 eV (neither too strong nor too weak). For pure Pt, Δ*E*_H*_ is *ca.* −0.2 eV (moderately strong binding).^65^ Meanwhile for pure Ni, Δ*E*_H*_ is approximately −0.8 eV (excessively strong binding). Notably, theoretical calculations predict that at a 50 : 50 Pt–Ni ratio, the hydrogen adsorption energy Δ*E*_H*_ approaches −0.1 eV, a value closer to the ideal value (thermoneutral condition, Δ*E*_H*_ = 0 eV) that favors faster adsorption–desorption kinetics. Despite this value is derived from DFT calculations and does not correspond to direct electrochemical measurements, however, experimental studies have corroborated the enhanced catalytic activity of Pt–Ni alloys in HER and ORR, demonstrating that Pt_3_Ni (111) surfaces exhibit significantly higher activity than pure Pt ^66^ , and that Pt–Ni nanoparticles display improved CO tolerance and hydrogen oxidation activity compared to monometallic Ni catalysts.^67^

The electronic and chemical properties of the Ni-noble metal alloys are also influenced by the surface properties of the composition of the composite materials. Computational analysis revealed that the d-band center (*ε*_d_), evaluated through multiple methods, shifts upward as the concentration of noble metal doping increases and as the crystalline structure becomes more ordered (Fig. 5d). This will modify the catalytic reaction over the Ni alloy catalyst system in application.

## 4. Surface chemistry alteration by alloying

While lattice geometry is modified upon alloying, the surface chemistry undergo similar process. This is the result of the process of lattice mismatch between the host and the dopant material, inducing strain or stress in the lattice. The impact of this process is tensile strain on the surface increases the availability of active sites and adjusts the electronic states to favor particular reaction process. For example, in Ni–Pt alloy, a computational models predict that a 3–5% strain alters the electronic structure and enhances oxygen reduction reaction (ORR) activity.^68^ Despite this finding pertains specifically to ORR and not directly to the hydrogen evolution reaction (HER), nevertheless, the mechanistic insight remains relevant, as strain engineering is a general strategy for tuning catalytic performance in alloy systems. Other unique phenomenon observed is when the Ni–Pt alloy growth on graphene substrate, DFT models show that depositing Ni–Pt alloys on a conductive graphene substrate enhances electron mobility and strengthens charge transfer effects. In addition, the synergy between Ni, Pt, and graphene amplifies HER activity by further lowering activation energy.^69^ In different study, it is reported that the lattice strain induced by combining Ni with noble metals adjusts surface reactivity as tensile strain in Ni–Pd systems reduces the energy required for bond cleavage in hydrodeoxygenation reactions.^70^ Alloying can induce lattice distortions on the surface and create new active sites. For example, the Ni–Mo alloy shows improved hydrogen evolution reaction (HER) activity due to lattice distortions and increased interfacial activity.^71^ The formation of ultrathin alloy shells, such as Pd–Ni, increases the utilization efficiency of metal atoms and enhances catalytic activity by maintaining a desirable surface structure.^72^

**Fig. 6:**
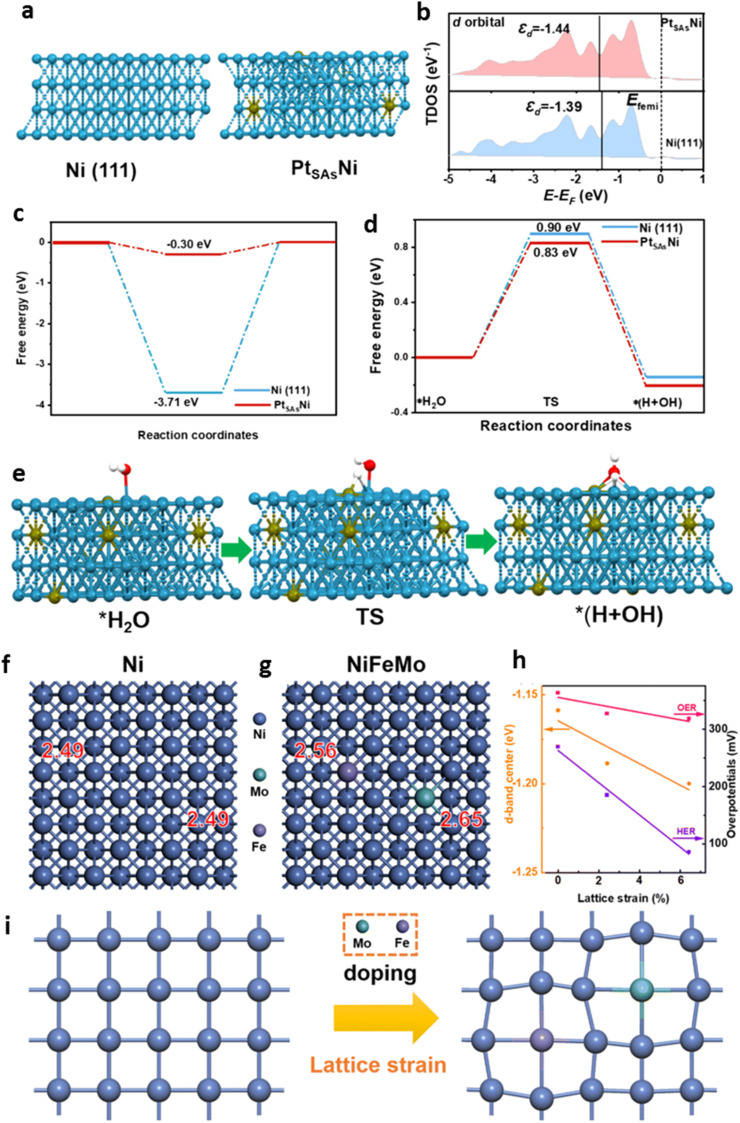
Schematic picture of Ni (blue) lattice distortion under Pt single atoms (SAs) (yellow ball) doping analysed using density functional theory (DFT) (a). (b) Comparison of TDOS for Ni (111) and Pt_SAs_Ni system. Corresponding Gibbs free energy (Δ*G*_H*_) for Ni (111) and Pt_SAs_Ni system (c). Comparison of reaction coordination and energy barriers for Ni (111) and Pt_SAs_Ni system in 1.0 M KOH as well as HER reaction pathway for Pt_SAs_Ni system (e). Hydrogen and oxygen represented by white and red balls. (f) and (g) are model for Ni and Mo and Fe co-alloyed Ni systems. (h) is the d-band center position and overpotential of NiFeMo system relative to lattice strain. (i) is schematic diagram of lattice strain in the presence of doping ((a–e) adapted from ref. 74, © 2024 Elsevier, and (f–i) adapted from ref. 73, © 2023 Royal Society of Chemistry).

The understanding of phenomenon of lattice strain in Ni upon alloying their effect on the surface chemistry properties have been further evaluated by introducing several metal dopant, *i.e.* Mn, Fe, Co and Cu.^20^ The alloying has effectively induced morphological changes related to lattice strain due to variations in the standard electrochemical reduction potential (Δ*E*°), mixing enthalpy (Δ*H*_mix_), surface energy alteration (Δ*γ*_o_), and chemical potential (Δ*µ*) between the dopant and the substrate metal. Analysis showed that lattice strain has changed the density of state, surface energy and chemical potential of the Ni, affecting the electrocatalytic activity. For example, Fe alloyed Ni, which has a negative mixing enthalpy and lower Fermi energy demonstrates excellence activity in oxygen evolution reaction (OER) with an overpotential (*η*) of 275 mV at a current density of 10 mA cm^−2^. Meanwhile, the Cu alloyed Ni, which has a positive mixing enthalpy exhibits high performance for hydrogen evolution reaction (HER) with an overpotential of 216 mV at 10 mA cm^−2^. This process is resulted from the lattice strain upon alloying, modifying the DOS of the catalyst and affecting the nature of reactant adsorption on the surface, influencing the catalyst preference activities. Fig. 6 shows typical effect of alloying on the Ni lattice modification and the surface chemistry that is represented by the change in the reaction coordinate and the d-band center as well as the reaction over potential related to lattice strain. As the figure reveals, the alloying induce lattice strain of nickel, modifying the d-band center and the surface chemistry of the catalyst, enhancing the HER and OER catalytic activities.^73^

As discussed earlier, Ni, as a transition metal, possesses a partially filled d-orbital, which is pivotal for its adsorption properties. The d-band center reflects the average energy level of the d-electrons in Ni, and variations in this energy state can directly impact the electronic properties of the surface. Changes in the d-band center alter the density of electronic states at the Fermi level, significantly influencing the metal's ability to interact with and adsorb various analytes.^75^ For example, Ramos-Castillo *et al.* evaluated theoretically how Ni that is alloyed with several transition metal influences their surface chemistry properties by evaluating the glycerol molecules adsorption and reactivity.^75^ They found that there is a significant correlation between the magnetic moment and d-band splitting in various Ni alloy systems. Alloying Ni with elements like Fe and Mn enhanced magnetism by providing unpaired electrons, whereas metals with filled d-bands, such as Cu and Zn, reduced this property. Adsorption of glycerol molecules analysis found that Ni_3_X surfaces, where X is Fe, Mn, Cu, Zn and V, revealed that lower d-band occupancy correlates with higher adsorption energy due to increased orbital hybridization between the metals and the OH groups in glycerol. The interaction with other molecules, *i.e.* dihydroxyacetone (DHA), was particularly dependent on the nature of the metals in the Ni_3_X catalysts. For example, Ni_3_Fe, with its partially filled d-bands, exhibited stronger binding with DHA due to enhanced orbital overlap with the partially filled 2p orbitals of DHA. In contrast, Ni_3_Co, with filled d-bands, did not exhibit such favorable orbital overlap, resulting in weaker binding and reactivity with DHA.

Structural modifications occurring on the surface of the Ni catalyst lead to alterations in the active sites, thereby governing the selectivity and catalytic activity of the process. Furthermore, modification of the catalytic activity and selectivity alter the reaction pathways during the catalytic process, which can be associated with the tailored electronic structures, optimizing the adsorption and activation of reactants, and selective activation of reactant and spill-over. For instance, the incorporation of indium into Ni–Ga alloys creates electron vacancies that enhance the catalytic reaction rates,^46^ optimizing the adsorption and activation of reactant. In other case, noble metals in single-atom alloy (SAAs) can facilitate the selective activation of reactants and the spillover of adsorbed species onto the Ni surface, where the main catalytic reactions occur.^76,77^ This mechanism enhances the overall efficiency and selectivity of the catalytic process.^76^ An enhanced specific reaction pathway has also been observed in Pd–Ni alloy surfaces. Alloying between these two metal exhibit significantly improved selectivity for hydrogenation reactions compared to pure Pd catalysts.^72^ The presence of noble metals can also modify the reaction pathways by altering the adsorption energies of intermediates, as seen in Pt–Cu–Ni ternary alloys, which optimize the kinetics of methanol oxidation reactions.^78^ The combination of Ni and noble metals creates a dual-active-site mechanism. In the case of Pt_1_@Ni encapsulated in nitrogen-doped carbon nanotubes (NCNT), the fast hydrogen spillover effect on Ni supports and accelerated water dissociation are observed. This dual mechanism significantly enhances HER performance in both acidic and alkaline media.^51^

An example of how alloying modify the reaction pathways in Ni is observed during CO_2_ hydrogenation to CH_4_ over Ni_4_Fe system under thermocatalytic reaction pathway.^79^ In Ni_4_Fe systems, the CO_2_ hydrogenation pathway proceeds initially through the formation of CO, which is subsequently hydrogenated to CH_4_. It has been known that the common reaction in CO_2_ hydrogenation involves two primary pathways, *i.e.* carboxyl (*COOH) and formate (*HCO), initiated with CO_2_ adsorption *via* either the O-terminal (*COOH formation) or the C-terminal (*HCOO formation). While direct CO_2_ dissociation to *CO and *O is also plausible, the resulting *CO may proceed through either the formate or carboxyl pathway. In this study, it is reported that the Ni_4_Fe alloy promotes CO_2_ dissociation and C–O bond cleavage in HCOO to form HCO more effectively than pure Ni.

**Fig. 7:**
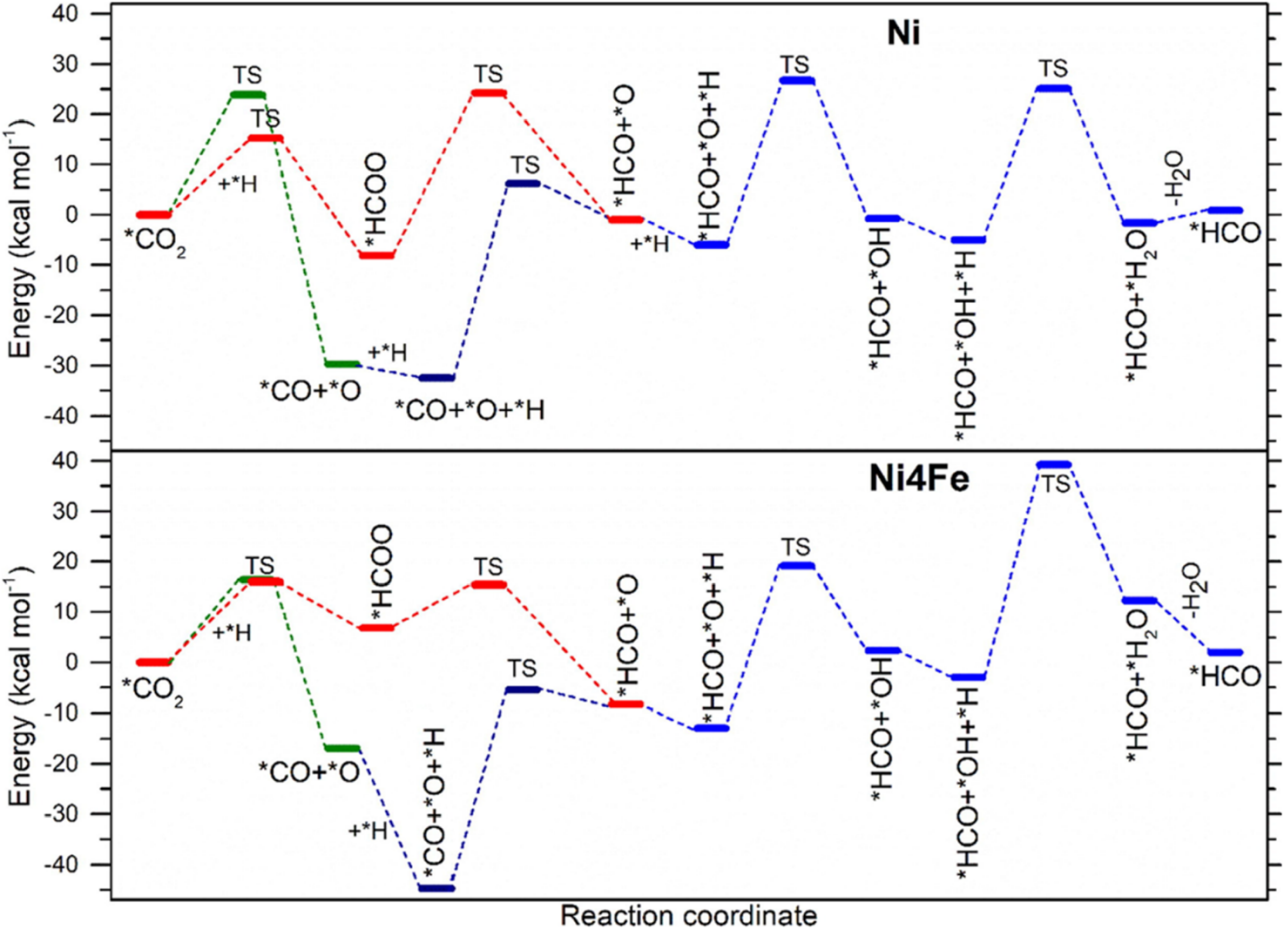
Energy diagram for the formation of *HCO *via* HCOO pathway and CO_2_ direct dissociation pathway on Ni (111) and Ni_4_Fe (111). TS is transition state (reproduced from ref. 79, © 2024 Elsevier).

Typical energy diagram for the formation of this *HCO *via* the *COOH pathway over Ni and Ni_4_Fe surfaces with partial contribution from direct *CO_2_ dissociation energy is presented in Fig. 7. As the figure shows, the activation energy for *COOH formation from *CO_2_ on Ni is lower (12.3 kcal mol^−1^) than *CO_2_ dissociation (23.9 kcal mol^−1^), whereas it was 20 kcal mol^−1^ and 16.3 kcal mol^−1^, respectively on Ni_4_Fe. This indicates that the NiFe alloy promotes *CO_2_ dissociation, reflecting critical modification of reaction pathways due to the alloying process. Regarding the *CO_2_ dissociation *via* the *COOH pathway, in this study, it is found that it is energetically favoured for the *CO_2_ → *CO → *HCO transformation on both surfaces with *HCO formation was identified as *CO + *H ↔ *HCO. However, due to the nature of instability of *HCO, it decomposes back to *CO, *via* the reverse water–gas shift (RWGS) reaction. Because of a strong binding energy of *CO to catalyst surface, nonetheless the calculation shows a better desorption on NiFe compared to Ni. This improves long-term stability of the Ni_4_Fe catalyst by mitigating active site blockage. On the other side, the energy calculation of HCO formation and subsequent CH_4_ production on Ni and Ni_4_Fe alloy catalysts indicates a higher formation rate of HCO on the alloy surface. The result also shows that HCO decomposition to CH and O proceeds with similar activation energies on both surfaces (*E*_a_ ≈ 23 kcal mol^−1^). Calculation also indicates that the alloy promotes the formation of CH and O more effectively than the Ni system. Regarding the CH_3_ hydrogenation to CH_4_, the activation energy (*E*_a_ ≈ 26 kcal mol^−1^) is similar on both surfaces. Thus, this fact further demonstrates how alloying effectively modify the reaction pathways for enhanced catalytic reaction.

Regarding the CO_2_ reduction in Ni-based alloy, while tunes the electronic structure alloying also reshapes the competitive balance between HER and CO_2_RR. For example, Ni–Cu and Ni–Bi alloys favor CO formation by stabilizing *CO intermediates, whereas Ni–Sn and Ni–In systems enhance HCOO^−^ production through weakened *H adsorption and facilitated proton-coupled electron transfer. Hydrocarbon formation (*e.g.*, CH_4_, C_2_H_4_) has been observed in Ni–Cu alloys under high current densities, highlighting the role of alloying in extending product pathways beyond simple CO. Quantitative metrics demonstrate that alloying shifts selectivity. Ni–Bi alloys achieve faradaic efficiency (FE) values exceeding 80% for CO at moderate overpotentials, while Ni–Sn alloys show high FE for formate (>70%). Partial current densities scale with alloy composition, reflecting the density of stabilized active sites. These data underscore the need to evaluate CO_2_RR performance not only by onset potential but also by product-specific current densities.

The mechanistic determinant for the CO_2_RR reaction generally is follow: firstly, as alloying modulates the d-band center, it tunes *CO adsorption. Stronger binding favors CO stabilization but risks site blocking. Meanwhile, weaker binding promotes desorption and selectivity toward formate. Secondly, suppression of *H adsorption is critical to reduce HER competition. Alloying with Bi or Sn weakens *H binding, thereby enhancing CO_2_RR selectivity. Thirdly, alloying alters charge redistribution at the interface, influencing proton availability and electron transfer kinetics. Fourth, electrolyte composition and cation identity (*e.g.*, K^+^*vs.* Na^+^) modulate double-layer structure, shifting selectivity between CO and formate. Finally, Ni alloys that destabilize *H while stabilizing *CO intermediates effectively suppress HER, enabling higher CO_2_RR efficiency.

**Fig. 8:**
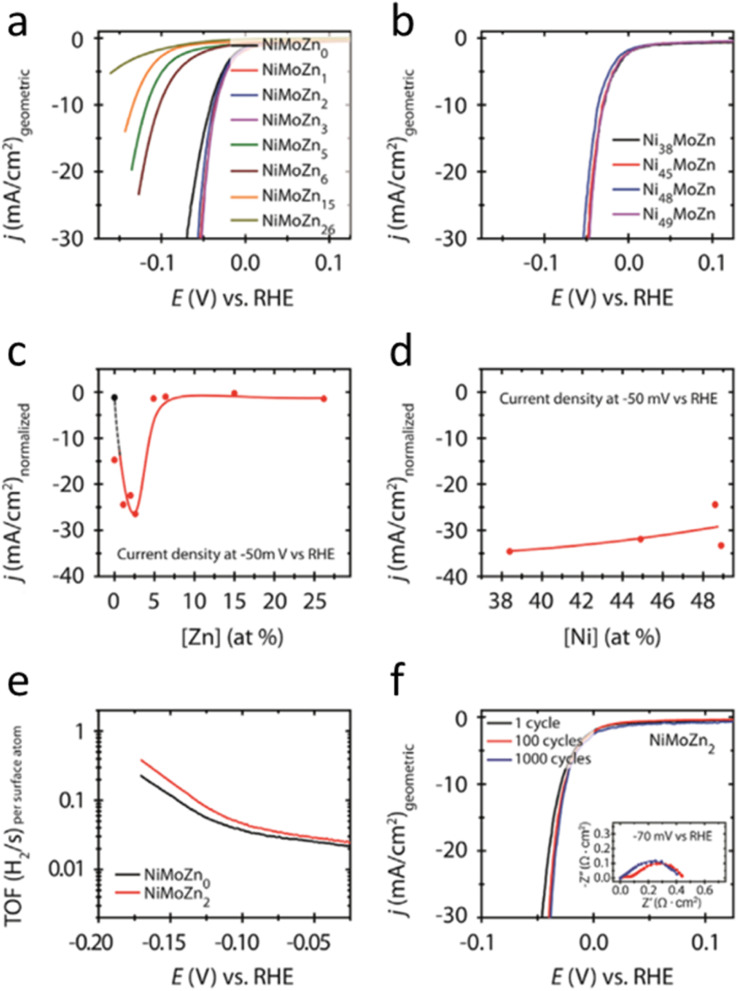
(a) and (b) are iR-corrected CV curves in 0.5 M H_2_SO_4_ electrolyte for high-loading NiMoZn alloy nanoparticles with varying Zn and Ni levels, respectively. (c) and (d) highlight how Zn and Ni concentrations affect current density at 50 mV *vs.* RHE. Dots represent experimental data; lines (solid and dashed) are guides. Pure Ni NPs are shown as a black dot in (c). (e) Shows the turnover frequencies (TOF) of NiMoZn_0_ and NiMoZn_2_, based on cathodic CV sweeps. (f) Presents the stability of NiMoZn_2_, with iR-corrected cathodic sweeps from the first cycle, and after 100 and 1000 cycles. The inset shows Nyquist plots at 70 mV *vs.* RHE, with ohmic resistance removed for clarity (reproduced from ref. 85, © 2015 Elsevier).

Nevertheless, as CO_2_RR often induces surface reconstruction and carbonate deposition, alloying may mitigate these effects by enhancing structural robustness and facilitating reversible adsorption–desorption cycles. Ni–Cu^80^ and Ni–Bi^81^ alloys, for instance, maintain stable product selectivity over extended electrolysis, highlighting the importance of alloy-induced stabilization. Such unique changes in structure and physical and chemical properties resulting from the alloying process is further shown in the following few examples where alloying Ni with other metals improves resistance to acid-induced corrosion and chemical attack.^82^ It has been well-acknowledged that the dissolution issue of Ni in acidic environments is critical. However, the alloying effect has effectively modified the crystal stability so that it improves their resistance to the corrosion or dilution under acidic environment.^83,84^ For example, NiMoZn ternary alloy in hydrogen evolution reaction (HER) indicated that the catalytic properties is favourable in acidic media and influenced by the concentration of Zn in the alloy. When the concentration of Zn is low the ternary catalyst facilitates the charge transfer process, enhancing the intrinsic electrocatalytic performance. Meanwhile, when the Zn concentration is high the proton adsorption on the electrocatalyst surface is inhibited, reducing HER efficiency.^85^ The optimal Zn concentration in NiMoZn alloy is 1–3 atomic percent (at%) Zn (Fig. 8). This condition shows significantly higher HER activity compared to most non-noble metal electrocatalysts. The NiMoZn alloy with 2 at% Zn exhibits excellent stability in acidic conditions, making it a promising candidate to replace noble metal electrocatalysts like platinum for water splitting processes. As surface chemistry is influenced by the surface structure, these results present as evidence how alloying effectively modifies the catalytic properties of Ni alloy system.

Enhanced activity an stability of HER as in the above condition (acidic media) also demonstrated by the different Ni alloys system such as NiMo–Mo_2_C/C hybrid, attributed to its anchored structure, the result of alloying process^86^ and NiMo polyhedron MOF, benefiting from the structural merits of MOF and the synergy between Ni and Mo.^87^ Ni–Ru–Pt alloy also exhibits special HER activity.

**Fig. 9:**
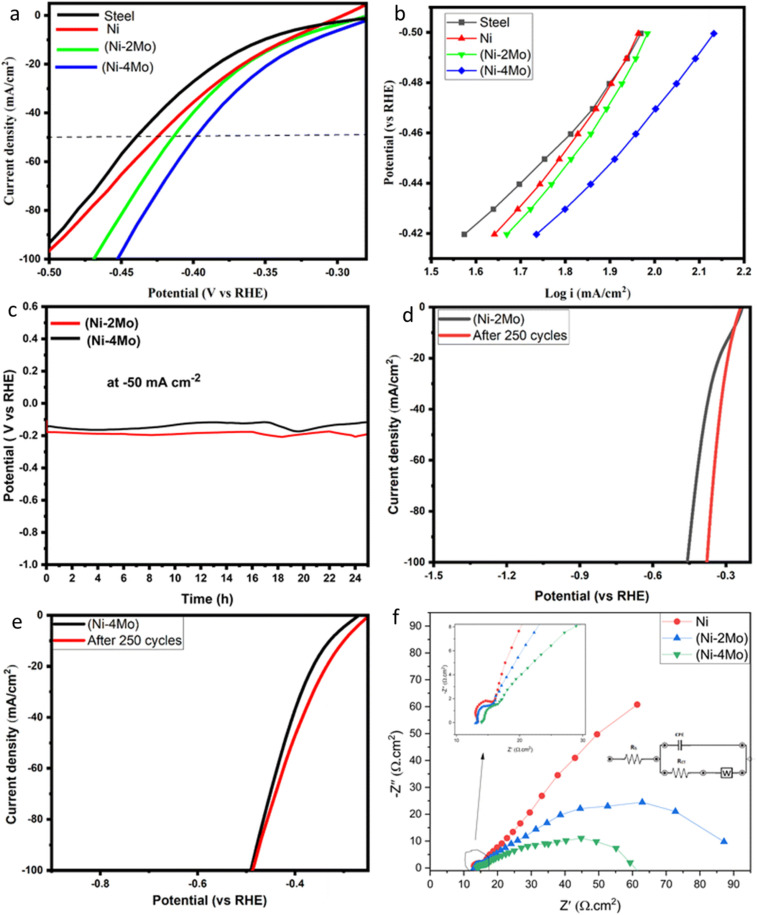
(a) Polarization curves of the hydrogen evolution reaction on the deposited electrocatalytic materials in mol L^−1^ H_2_SO_4_ at 298 K. The potential scan rate is 50 mV s^−1^. (b) Tafel plots of hydrogen evolution reaction on the deposited electrocatalytic materials in mol L^−1^ M H_2_SO_4_ at 298 K. (c) The chronopotentiometric curves for HER on the surface of Ni–2Mo and Ni–4Mo catalysts at a current density of −50 mA cm^−2^. (d and e) LSV for Ni–2Mo fresh and after 250 cycles and Ni–4Mo fresh and after 250 cycles, respectively. The curves are collected in 0.5 mol L^−1^ H_2_SO_4_ at 25 °C with a scan rate of 50 mV s^−1^. (f) Measured (dots) and fitted (solid lines) Nyquist plots for Ni, (Ni–2Mo), and (Ni–4Mo) coatings in 0.5 mol L^−1^ H_2_SO_4_. Insets: the equivalent circuit for EIS fitting; the locally enlarged plots for showing semicircles (reproduced from ref. 88, © 2025 The Royal Society of Chemistry).

Other example of how alloying produce different structure in Ni–Mo alloys with cauliflower morphology and how their HER performance in acidic media depended on their alloying ratio. An example of the HER performance of the Ni–Mo alloys in acidic media, *i.e.* 0.5 M H_2_SO_4_ solution, is shown in Fig. 9.^88^ It is shown that incorporating Mo into a Ni matrix enhances HER activity, as indicated by a reductions in overpotential and augmented electrocatalytic activity (Fig. 9a). This study again posits a direct correlation between surface structure, molybdenum concentration and cathodic polarization, with the Ni–4Mo alloy (65.15% Mo) exhibiting demonstrably superior performance compared to its Ni–2Mo (56.21% Mo) counterpart. Analysis highlighted that this improved activity is attributed to the special surface chemistry and partially filled d-orbitals of Mo, facilitating hydrogen adsorption, weakening hydrogen bonding to the catalyst surface, and ultimately promoting water splitting.

Electrochemical analysis at 50 mA cm^−2^ reveals a lower overpotential for Ni–4Mo (−390 mV) in comparison to steel, nickel, and Ni–2Mo, signifying improved electrocatalytic performance (Fig. 9a), providing quantitative evidence of electrocatalytic activity dependence on the alloying condition. Tafel plot analysis confirms this, showing a smaller Tafel slope (−113 mV dec^−1^) and higher exchange current density (1.250 mA cm^−2^) for Ni–4Mo, indicative of faster HER kinetics (Fig. 9b). Chronopotentiometry reveals that the Ni–4Mo has a better stability and greater efficiency if compared to Ni–2Mo (Fig. 9c). Linear sweep voltammetry shows comparable initial activity and reasonable stability for both Ni–4Mo and Ni–2Mo, but subtle degradation suggests phase transformations requiring further investigation (Fig. 9d and e). Finally, electrochemical impedance spectroscopy corroborates Ni–4Mo's superior HER activity through its lowest charge transfer resistance, signifying enhanced electron transfer during the reaction (Fig. 9f). Overall, the study highlights how the alloying condition determines the catalytic activity of the Ni alloy system.

**Fig. 10:**
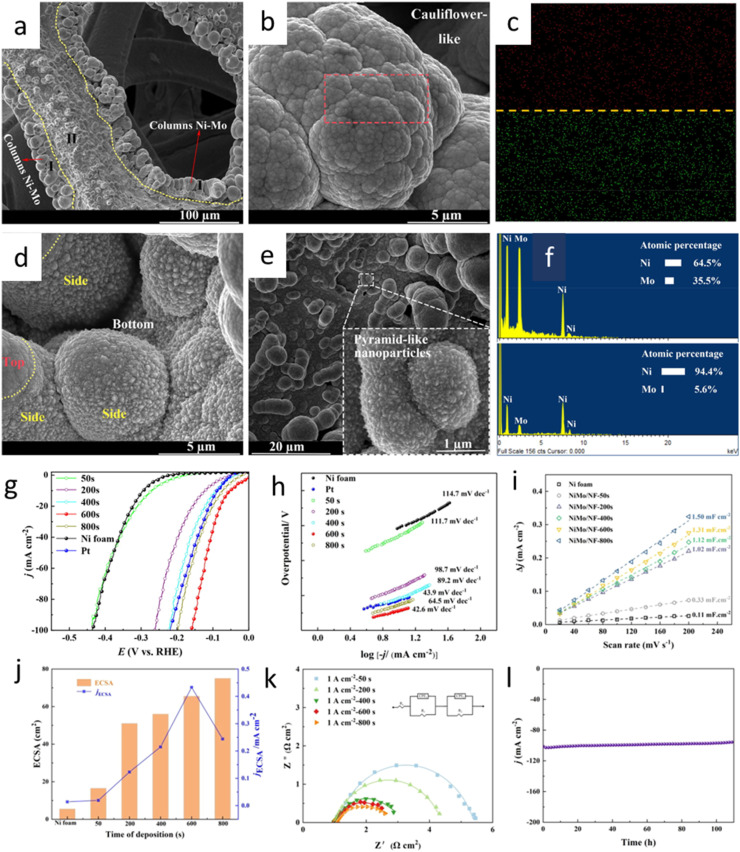
Detailed properties of the Ni–Mo/NF electrocatalyst. (a) Scanning electron microscopy (SEM) images showing the Ni–Mo/NF skeleton. (b) A magnified view depicting the top area of the columnar structure. (c) Elemental mapping showing the distribution of nickel (top) and molybdenum (bottom) within the structure. (d and e) High-resolution SEM images highlighting the presence of pyramid-like nanoparticles on the cylinder sides and bottom of the columnar region. (f) Energy-dispersive X-ray spectroscopy (EDS) analysis indicating the elemental composition of different particles. (g) Linear sweep voltammetry curves for Ni–Mo/NF samples deposited for varying durations; (h) corresponding Tafel plots; (i) capacitive current densities plotted against scan rates; (j) electrochemical surface area (ECSA) and normalized ECSA (jECSA) values at an overpotential of −0.150 V *vs.* RHE; (k) Nyquist plots derived from electrochemical impedance spectroscopy; and (l) chronoamperometry data illustrating the catalyst's long-term stability (reproduced from ref. 89, © 2022 Elsevier).

Fig. 10 shows interesting example of how structure in Ni alloyed with Mo can influences the surface chemistry of the catalyst.^89^ It is shown how the deposition time influences the resulting nanostructure morphology and, consequently, the electrochemical performance as well as the HER activities of the fabricated electrodes. Morphological analysis, detailed in Fig. 10a–f, highlights the formation of distinct nanostructures, namely cauliflower- and nanopyramid-like morphologies, with varying Ni : Mo ratios. The optimized Ni–Mo film, deposited for 600 seconds, exhibited a remarkably low overpotential of 53 mV at 10 mA cm^−2^, surpassing the performance of both Pt and bare Ni foam electrodes (Fig. 10g). This superior performance is further corroborated by the low Tafel slope of 42.6 mV dec^−1^, indicating favorable reaction kinetics and suggesting a Volmer–Heyrovsky mechanism (Fig. 10h).

Electrochemical measurements, including electrochemically active surface area (ECSA) analysis and electrochemical impedance spectroscopy (EIS), were conducted to delve into the underlying mechanisms. The ECSA results revealed a positive correlation between deposition time and the density of active sites, peaking at the 600 second deposition mark. While subsequent deposition led to a sustained high active site count, the increased overpotential at 800 seconds was attributed to a mushroom-like structure hindering bubble release, demonstrating a critical relationship between morphology and performance (Fig. 10i and j). The EIS analysis, modeled with a two-time constant series circuit (Fig. 10k), revealed a significant decrease in charge transfer resistance with increasing deposition time up to 600 s, signifying improved interfacial reaction kinetics. The synergistic effect of enhanced active sites and facilitated charge transfer resulted in a maximal ECSA-normalized current density (jECSA) for the 600 s sample, further validating its superior HER catalytic activity. Finally, the study demonstrated robust long-term stability of the optimized sample, with minimal current density fluctuation observed during extended operation (Fig. 10l). This underscores the practical potential of the developed Ni–Mo/NF electrode for efficient and stable HER.

However, when Ni is alloyed with Sn (Ni–Sn Alloy), the enhanced activity and stability was demonstrated under alkaline media. This is interesting and attributed to its lower hydrogen overpotential compared to Raney-Ni and nickel net electrodes.^90^ However, for the case of NiMo alloy, despite in above mentioned case where it is active in acidic media, changing the preparation method (sputtering) and catalyst morphology (nanorods shape), the NiMo alloy system showed excellent activity during HER in alkaline media. This is associated with its vertical nanorod structure and the synergistic interaction between Ni and Mo.^91^ Similar process was also observed in NiAg, where the result of the morphological alteration (flat and smooth surface) and alloying that creates metastable alloy realizes optimal hydrogen binding sites.^92^ Moreover, Ni–Ru–Pt ternary alloy exhibits an enhanced hydrogen evolution reaction (HER) efficiency, achieving low overpotential and high turnover frequencies (TOFs) in both acidic and alkaline electrolytes.^93^ The ternary alloy shows great long-term stability, making it a promising candidate for electrochemical energy technologies. Table 1 compares the detailed properties of the Ni alloy system in the catalytic reaction application.

**Table 1:** Example of Ni-alloys system performance in HER and HOR reactions application

Alloy	Electrolyte	Overpotential	TOF	Stability	Key feature	Ref.
NiMoZn	0.5 M H_2_SO_4_	100 mV *vs.* RHE	∼0.22 s^−1^ per surface atom (at 100 mV *vs.* RHE)	Stable upto ∼1000 cycles	Optimal Zn boosts charge transfer	85
Ni–Sn	30 wt% KOH	137 mV *vs.* SHE	—	Stable for 15 days operated at 2000 A m^−2^	Lower overpotential than Raney-Ni	90
Ni–Mo nanorods	1.0 M KOH	48 mV	—	145 h at 10 mA cm^−2^	Efficient due to nanorod structure	94
NiMo–Mo_2_C/C hybrid	0.5 M H_2_SO_4_	90 mV	—	High stability under ∼3000 cycles	Anchored structure nhances activity	86
NiMo polyhedron MOF	0.5 M H_2_SO_4_	80 mV	—	Stable for 24 h continuous operation	Synergy between MOF and metals	87
Ni–Ru–Pt	0.5 M H_2_SO_4_/1.0 M KOH	22.14 mV	4.13 H_2_ s^−1^ at 50 mV/3.88 H_2_ s^−1^ at 100 mV	High stability under ∼3000 cycles	Ni alloying enhances HER activity	95

**Fig. 11:**
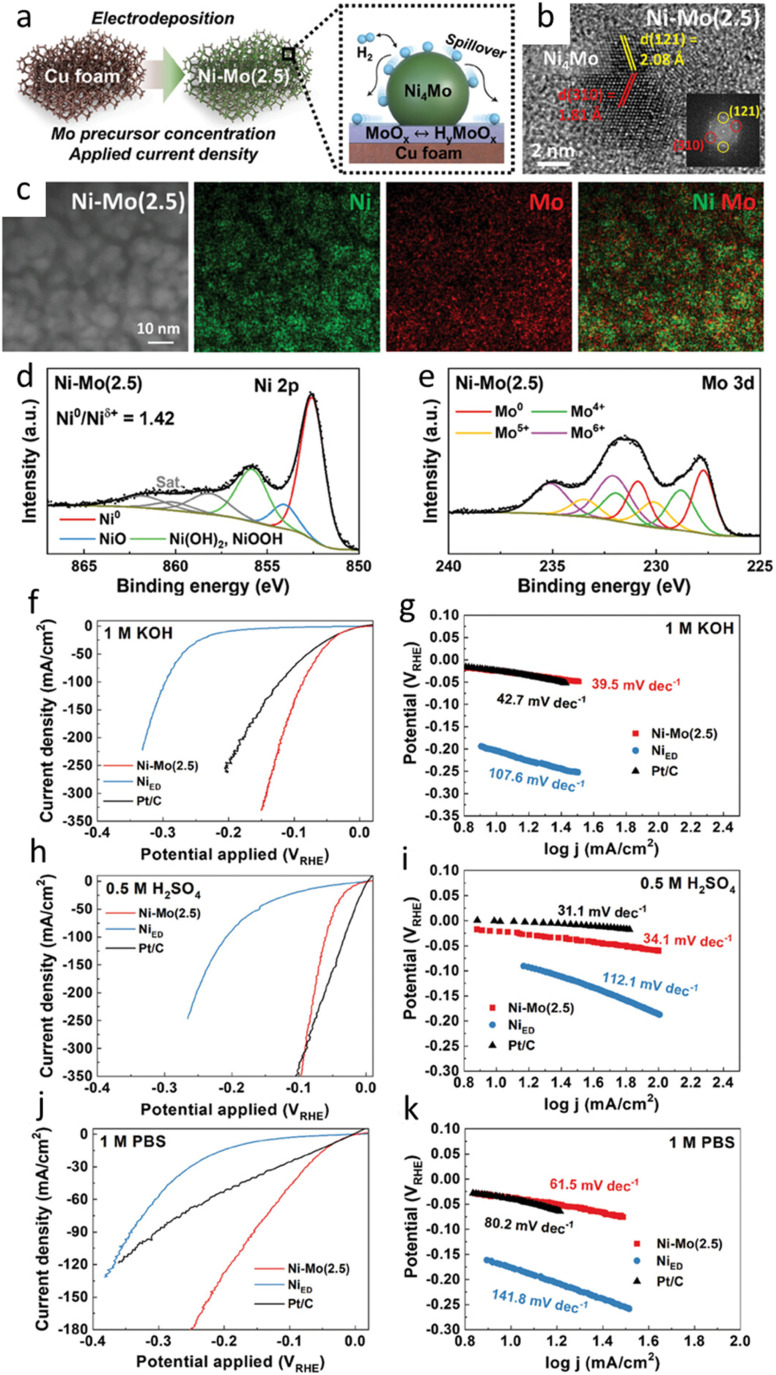
The structural and electrochemical properties of a Ni–Mo electrocatalyst. (a) A schematic representation of the Ni–Mo(2.5) catalyst, with an inset depicting its heterogeneous microstructure. (b) High-resolution transmission electron microscopy (HRTEM) image of the Ni–Mo nanoalloy within Ni–Mo(2.5), accompanied by a selected area electron diffraction (SAED) pattern (inset). (c) Focused ion beam-transmission electron microscopy (FIB-TEM) cross-sectional micrographs and corresponding energy-dispersive X-ray spectroscopy (EDS) elemental mapping of Ni–Mo(2.5). (d) X-ray photoelectron spectroscopy (XPS) spectra of the Ni 2p and (e) Mo 3d core levels for the electrodeposited Ni–Mo(2.5) catalyst. Hydrogen evolution reaction (HER) polarization curves and Tafel plots for Ni–Mo(2.5), Ni_ED_, and Pt/C catalysts measured in 0.1 M KOH (f and g), 0.5 M H_2_SO_4_ (h and i), and 1 M PBS (j and k) electrolytes. Linear sweep voltammetry (LSV) was performed at a scan rate of 2 mV s^−1^ with iR compensation (reproduced from ref. 96, © 2024 Wiley).

Nevertheless, as presented earlier in this review, Ni–Mo alloys system does promise special performance in many applications. Extensive study have been found to be dedicated to the evaluation of their performance in HER reaction. Song *et al.* investigates the performance of a Ni–Mo alloy as an electrocatalyst for the hydrogen evolution reaction (HER) across acidic, alkaline, and neutral pH conditions (Fig. 11).^96^ The catalyst, prepared using an electrodeposition technique on a copper substrate, exhibits a stoichiometric, homogeneous distribution of nickel and molybdenum (Fig. 11a–e). A key aspect of the preparation involves the formation of a MoO_3_ phase on the Cu substrate, which is proposed to enhance HER activity by facilitating hydrogen spillover.

The study demonstrates that Ni–Mo(2.5) displays superior HER activity in alkaline media compared to commercial Pt/C, requiring lower overpotentials to achieve comparable current densities (Fig. 11f and g). In acidic and neutral electrolytes, its performance is either comparable to or exceeds that of Pt/C, especially at higher current densities (Fig. 11h–k). The improved activity is attributed to the intrinsic catalytic properties of the alloy rather than an increased electrochemical surface area. Tafel plot analysis suggests that water dissociation is the rate-determining step in alkaline and neutral conditions, whereas the HER mechanism follows the Volmer–Tafel pathway in acidic media and the Volmer–Heyrovsky pathway in alkaline/neutral electrolytes. Regarding the catalyst stability, the Ni–Mo(2.5) exhibits excellent stability, outperforming both electrodeposited Ni and Pt/C in long-term durability tests. Post-analysis using SEM and XPS confirms the structural and chemical integrity of the catalyst after extended operation, although surface oxidation is observed. In alkaline water electrolysis, the catalyst demonstrates excellent cell performance with minimal overpotentials compared to other Ni–Mo based electrocatalysts. Thus, it is suggested that Ni–Mo(2.5) is a promising alternative to Pt/C for HER, particularly in alkaline conditions, owing to its superior activity, stability, and cost-effectiveness.

One thing to be noted that while core-level shifts observed in XPS spectra are frequently cited as evidence of charge transfer in alloy systems (*e.g.*, Ni–Pt), binding energy shifts reflects the presence of final-state effects, oxidation state changes, or differential charging. However, the XPS alone cannot unambiguously prove electronic redistribution and explained the overall mechanism. Instead, XPS should be interpreted alongside complementary techniques to obtain a complete figure of the process.

Important to be noted that the modification of the catalytic activity of Ni-based alloys does not merely related to the alteration of electronic properties but also due to presence dynamic surface phenomena. Under realistic electrochemical conditions, the active phase is often not the pristine alloy surface but a reconstructed oxyhydroxide layer. For example, during OER, Ni undergoes potential-driven reconstruction into NiOOH/Ni(OH)_2_, with alloying elements such as Fe, Mo, or Cr segregating to the surface and stabilizing these oxyhydroxide phases. This surface segregation alters local coordination environments and electronic states, thereby redefining the true catalytic interface. Thus, while bulk alloying tunes the electronic structure, the catalytic activity is ultimately governed by the reconstructed surface phase.

The following phenomena describe these processes. In alkaline HER, the Volmer step (water dissociation) is often rate-limiting. Alloying that lowers the barrier for O–H bond cleavage (*e.g.*, Ni–Mo, Ni–Pt) enhances kinetics by optimizing *H adsorption and facilitating proton/electron transfer. In OER, scaling relations between *OH, *O, and *OOH intermediates dictate overpotential. Alloying shifts adsorption energies, breaking unfavorable scaling and reducing the energy barrier for the *O → *OOH transition. Next, in CO_2_RR, the competition between *CO and *H adsorption is central. Alloying strategies that weaken *H coverage while stabilizing *CO intermediates suppress parasitic HER and promote CO_2_ reduction selectivity toward CO or formate. These mechanistic descriptors provide a direct link between alloy-induced electronic modulation and catalytic performance.

## 5. Structural modification by alloying

During the alloying process, modification of the lattice structure is inevitable. The modification of lattice geometry or structure is due to the existence of lattice mismatch between the two metals. In different process, alloying may also form interstitial growth where individual metal grow into their crystalline domain in the bulk crystal or under a certain condition, the foreign metal may coat the host metal forming core–shell structure. All the system will modify the electronics and chemical properties, producing unique new properties beneficial for catalysis application. For example, Ni–Pt alloying process produces a new surface geometry with a homogeneous distribution of active sites, improving the stability and durability in fuel cell applications.^49^ In the case of core–shell structure, it allows precise control over surface composition where the metal shell provides special catalytic activity, while the Ni core modifies the electronic structure through strain and electronic effects. Ni–Pd core–shell structure nanoparticles demonstrated this unique performance in ethanol oxidation reactions for fuel cell applications.^97^

**Fig. 12:**
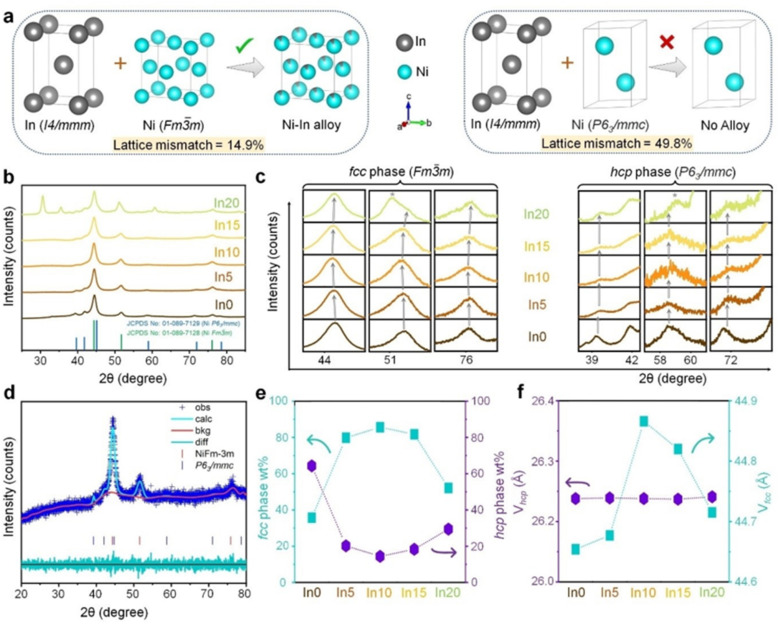
The formation and structural characteristics of Ni–In heterogeneous alloys. (a) A schematic illustration of the alloy formation process, influenced by lattice mismatch between tetragonal In and the face-centered cubic (fcc) and hexagonal close-packed (hcp) phases of Ni. Crystal structures were derived from Rietveld analysis of X-ray diffraction (XRD) patterns. (b) XRD patterns of the Ni–In alloys juxtaposed with standard reflections from the Joint Committee on Powder Diffraction Standards (JCPDS) files for fcc and hcp Ni. (c) Expanded views of specific regions from panel (b) reveal a progressive shift in fcc reflections with increasing In concentration, while hcp reflections remain stable. (d) A Rietveld-refined XRD pattern of In5 is shown, including observed (Obs) and calculated (Calc) patterns, the difference plot (diff) between them, and the background plot (bck). (e) The relative weight fractions of the fcc and hcp phases are plotted as a function of In atomic percentage (at%) in the Ni–In alloys. (f) The variation in unit cell volume for both fcc and hcp phases is depicted as a function of In at%, with parameters for panels (e) and (f) obtained through Rietveld refinement of the XRD data (adapted from ref. 98, © 2023 Wiley-VCH GmbH).

Fig. 12 shows typical phenomenon of alloying Ni with other metals (In) that modify the structure and catalytic properties.^98^ As has been mentioned earlier, the origin of the structure modification is the existence of lattice mismatch between the Ni and In. Tetragonal indium (In) and face-centered cubic (fcc) nickel (Ni) has lattice mismatch as high as 14.9%, while the mismatch with hexagonal close-packed (hcp) Ni is 49.8% (Fig. 12a). The incorporation of In leads to phase transformation in Ni due to the increases in the fraction of the fcc phase in the Ni–In alloy at the expense of the hcp phase. For example, an alloy with 5 at% In (In5) can have an fcc phase close to 80% (Fig. 12d–f). The assimilation of In, which has a larger atomic radius, increases the fcc lattice volume up to a certain In concentration (In10), beyond which it drops. The unit cell parameters and volume of the hcp phase remain largely unaltered. Furthermore, the close association of Ni and In within self-assembled structures results in a distribution of lattice strain and defects at the atomic scale.

While lattice geometry changes with the alloying, the chemical properties of the Ni alloy experience a drastic modification. As the result of charge transfer from In to Ni, the metallic state of Ni (Ni0) is stabilized and In^(*δ*+)^ sites, which favors *OH adsorption is generated. This phenomenon improves the HER performance, with In5 showing the best activity, achieving 153 mL h^−1^ H_2_ evolution at −385 mV. The enhanced HER performance is attributed to better reactant binding on surface active sites in alkaline medium. The In5 alloy also demonstrates more spontaneous water adsorption and dissociation, with a lower activation energy barrier for the water dissociation step compared to other compositions. Furthermore, due to an optimal Δ*E*_OH_, In5 system helps balance the water dissociation process and prevents catalyst poisoning.

## 6. Mechanistic framework for catalytic activity improvement

It is important to distinguish between chemical, thermochemical, and electrochemical catalysis in the context of Ni-based alloys. Chemical catalysis generally refers to reactions occurring under ambient or moderate conditions where Ni facilitates bond rearrangements, such as hydrogenation of unsaturated hydrocarbons, by lowering activation barriers through adsorption and orbital interactions. Thermochemical catalysis involves reactions driven by elevated temperatures, where Ni's structural stability and resistance to sintering are critical—for example, in steam methane reforming or the Sabatier reaction for CO_2_ methanation. In these processes, heat provides the driving force for bond cleavage and formation, while alloying strategies mitigate deactivation from coking or sulfur poisoning. Electrochemical catalysis, by contrast, harnesses applied potential to drive redox reactions at the electrode–electrolyte interface. Here, Ni-based alloys play a pivotal role in tuning adsorption energies and charge-transfer kinetics for reactions such as the hydrogen evolution reaction (HER), oxygen evolution reaction (OER), and CO_2_ reduction. Alloying modifies the d-band center, orbital hybridization, and interfacial charge distribution, thereby directly influencing electron transfer pathways and catalytic turnover.

For a detailed expression, nickel undergoes profound transformations in its electronic structure, surface chemistry, and lattice configuration during alloying, and these changes directly influence catalytic performance across hydrogen evolution (HER), oxygen evolution (OER), CO_2_ reduction, and hydrocarbon reforming. To unify these diverse observations that have been discussed above, we generalize them into two overarching mechanisms: (i) ligand, strain and ensemble effects, and (ii) interfacial activation and double-layer control.

For the ligand, strain and ensemble effect, the process of alloying can be understood as follow: firstly, alloying introduces charge transfer (electronic redistribution) between Ni and the secondary metal, shifting the Ni d-band center and modifying adsorption energies. This is also be interpreted as the ligand effect. For example, in Ni–Bi alloys, XPS analysis shows binding energy shifts in Ni (2p) and Bi (4f), evidencing electron redistribution. This optimizes the adsorption strength of intermediates, enhances methanol oxidation activity, and mitigates CO poisoning by weakening Ni–CO bonds (Fig. 1 in manuscript). Secondly, alloying often induces lattice distortion or compression strain, which alters orbital overlap and conductivity. In Ni–Bi systems, for example, lattice compression improves electron/mass transfer, while in Ni–Fe layered double hydroxides (LDH), incorporation of FeOOH nanoparticles creates local strain fields that lower OER overpotentials. Strain effects thus directly accelerate reaction kinetics. Finally, alloying changes the spatial arrangement of active sites, creating new ensembles that favor multi-step reactions. In Ni–Fe alloys embedded in N-doped carbon nanoboxes, ensemble effects facilitate intermetallic charge transfer, downshift the Fe d-band center, and reduce OER/ORR barriers. Similarly, Pt–Ni alloys exploit ensemble geometries where negatively charged Pt and positively charged Ni synergistically interact with reactants, boosting HER activity. In general, ligand, strain, and ensemble effects collectively tune adsorption energies—balancing reactant activation with product desorption—and thereby enhance catalytic turnover.

For interfacial activation and double-layer control, the new properties due to alloying can be understood as follow: firstly, charge redistribution at interfaces: alloying modifies interfacial charge density, which governs water activation and adsorbate scaling. In Ni–Fe–La ternary alloys, La incorporation optimizes d-orbital hybridization with oxygen 2p orbitals, strengthening oxygen intermediate adsorption while lowering the rate-determining step barrier. This results in high OER activity (1 A cm^−2^ at 1.58 V) and long-term stability (>600 h). Secondly, orbital hybridization and double-layer modulation: recent advances in Ni-based layered double hydroxides (LDHs) highlight how orbital hybridization and interfacial modulation can dramatically enhance electrocatalytic performance. In particular, Cr-incorporated NiFe LDHs demonstrate that introducing Cr alters the spin state of Fe, leaving partially unoccupied *e.g.* orbitals that strengthen d–d orbital interactions. This orbital hybridization shifts the d-band center closer to the Fermi level, optimizing adsorption energies of oxygen intermediates and lowering the reaction barrier. Simultaneously, the layered double hydroxide structure facilitates extensive surface reconstruction, effectively modulating the electrochemical double layer and stabilizing active sites during operation. As a result, the Cr–NiFe LDH catalyst achieves low overpotential and long-term durability in oxygen evolution reaction (OER), underscoring the synergistic role of orbital hybridization and double-layer modulation in driving high activity. This example illustrates how electronic and interfacial engineering, beyond conventional alloying, can be harnessed to design robust Ni-based catalysts for sustainable energy conversion.^99^ Finally, interfacial reconstruction and adsorbate scaling: Ni–Au alloys exemplify how alloying induces structural transformation (orthorhombic uranium silicide-type structure) that alters band structure and adsorption scaling. This reconstruction tunes adsorption energies, lowering barriers for CO_2_ reduction and proton reduction. In general, interfacial activation and double-layer control regulate water activation, adsorbate binding, and stability under operating conditions, ensuring both high activity and durability.

This structured framework consolidates diverse case studies (Ni–Bi, Ni–Fe, Ni–Fe–La, Ce-NiFe LDH, Ni–Au) into mechanistic hypotheses that can guide rational design of Ni-based alloys for electrocatalysis.

## 7. Ni-based alloys synthetic strategy

The preparation technique to obtain the Ni alloys system is key to the exceptional performance in the HER or OER applications. Each fabrication route offers unique opportunities to tune ordering, segregation, strain, defect density, and interfacial chemistry—parameters that directly control electronic states, adsorption energies, and stability. Selecting appropriate method may facilitate the preparation of controlled stoichiometry Ni based alloys system, which is vital for specific application. Several methods have been demonstrated to achieve controlled properties Ni alloys system. In this section, we highlight how common synthesis strategies act as mechanistic strategy to achieve unique and novel properties of Ni-based alloys rather than generic preparation methods.

### 7.1. Electrodeposition and galvanic techniques

Galvanic replacement is amongst the most employed method to modify the Ni system with other metals, particularly noble metal. Galvanic replacement is a process where a less noble metal is replaced by a more noble metal when treated with a solution containing the latter in ionic form.^100^ In the context of noble metal modified Ni wires, the mechanism of galvanic replacement involves the spontaneous replacement of surface layers of Ni by a more noble metal, such as Pt, when treated with a solution containing Pt ions.^101,102^ The reaction is driven by the difference in the equilibrium potential of the two metal/metal ion redox couples.^100^ Galvanic replacement involves the spontaneous replacement of surface layers of a metal by a more noble metal when treated with a solution containing the latter in ionic form. The resulting bimetallic material can have a more noble metal-rich shell and a metal-rich core, leading to a decrease in noble metal loading and modification of its properties by the underlying metal.^100^

It is important to be noted that either electrodeposition and galvanic replacement provide atomically precise control over alloy composition and distribution. By adjusting deposition potential, electrolyte composition, or sacrificial templates, these methods enable ordering and segregation control, selectively enriching alloying elements at the catalyst surface. Such segregation alters adsorption energies and mitigates poisoning effects (*e.g.*, CO adsorption). Moreover, electrodeposition inherently introduces defect density modulation, creating vacancies, grain boundaries, and nanostructured morphologies that serve as catalytic hot spots. Galvanic replacement further promotes interface charge redistribution, forming heterointerfaces that accelerate charge transfer and stabilize active sites. Together, these techniques allow mechanistic tuning of adsorption/desorption kinetics in HER and OER, while enhancing tolerance to poisoning and corrosion.

**Fig. 13:**
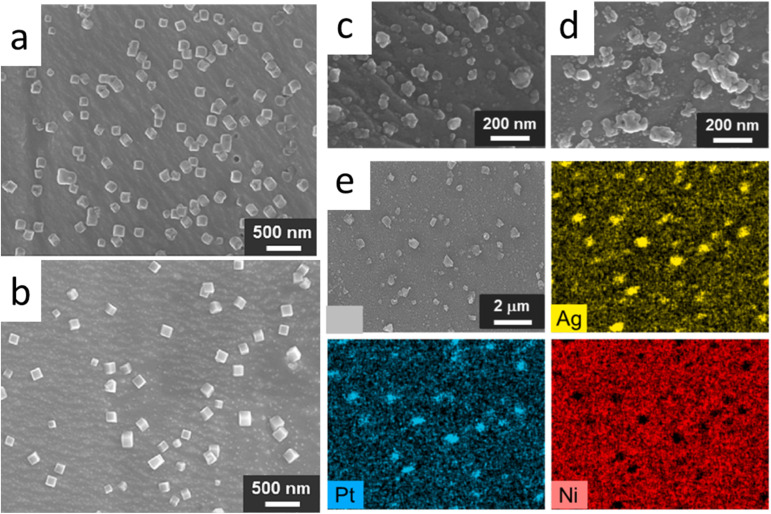
Field-emission scanning electron microscopy (FE-SEM) images of the surface morphology and the elemental analysis of Ni wires following treatment in aqueous solutions. (a) and (b) depict surfaces treated with 1.0 mM CuCl_2_ and 1.0 mM Cu(NO_3_)_2_, respectively, for 1 hour at 30 °C. Images (c) and (d) show the surfaces of a Ag/Ni wire electrode and a Pt-modified Ag/Ni wire electrode. The Ag/Ni wire electrode was produced by immersing Ni wire in a 1.0 mM AgNO_3_ aqueous solution for 10 minutes. Subsequently, the Pt-modified Ag/Ni wire electrode was fabricated by treating the Ag/Ni wire electrode in a 1.0 mM K_2_PtCl_4_ aqueous solution for 10 minutes. (e) Presents energy-dispersive X-ray spectroscopy (EDS) results for the Pt-modified Ag/Ni wire electrode, including a SEM image and elemental mapping for Ag, Pt, and Ni ((a and b) reproduced from ref. 103, © 2021 Elsevier; (c–e) are reproduced from ref. 102, © 2024 Elsevier).

Fig. 13 shows typical example of galvanic reaction process result for the preparation of Cu nanostructure on the Ni wires surface. It is achieved by immersing the Ni wire into the Cu^2+^ solution (*e.g.* CuCl_2_ and Cu(NO_3_)_2_) and by galvanic replacement process cubic-like nanostructure are formed on the surface of Ni wire (Fig. 13b). As presented in the Fig. 13b, the cubic nanostructure grows densely when CuCl_2_ solution was used. This could be the result of higher equilibrium potential difference with Ni wire in CuCl_2_ than the Cu(NO_3_)_2_. The cubic nanostructure is Cu_2_O. Fig. 13c–e shows another example of galvanic replacement process in growing AgPt bimetallic nanostructure on the Ni wires involving consecutive immersion of Ni in AgNO_3_ and K_2_PtCl_4_ solution. EDS analysis result confirm the formation of AgPt bimetallic phase growth on the Ni wires surface.

Galvanic replacement provides a convenient route to synthesize metallic films and nanostructures.^104^ It allows for the precise manipulation of the oxidative dissolution of sacrificial template metals and reductive deposition of alternate metals, leading to the fabrication of highly active catalysts.^105^ However, the process may be limited by the occurrence of side chemical and electrochemical processes in aqueous solutions, which can be prevented in non-aqueous media.^106^

Recent advancements in galvanic replacement reactions have focused on the controlled formation of nanostructures in non-aqueous media, which promotes the deposition of metallic nanoparticles on macro- and nanosurfaces of the substrate, allowing for the fabrication of nanomaterials with predetermined functional properties.^106^ The use of galvanic replacement in molten salts has been explored to design new compositions of layered transition-metal carbides, which can further be involved in etching processes to yield new 2D transition-metal carbides (MXenes).^107^

On the case of NiPd, a common method involves immersing Ni wire in an aqueous solution of tetrachloropalladate (PdCl_4_)^2−^. However, reproducibility issues were noted, which were mitigated by pretreating the Ni wire with 1.0 M HCl and increasing the solution temperature to 50 °C. This improved the control over Pd modification by adjusting the immersion time.^108^ Similar approach can also be applied in the case of NiAg. This method was found to be effective, although the amount of Ag deposited was limited to less than 100 nm in size.^103^ In other case, for example NiPt, by controlling the potential at relatively higher potential, for example −0.6 V *vs.* Ag/AgCl, electrodeposition, instead of galvanic reaction, will be occurred. In this case, multilayer NiPt or segmented nanowires will be formed.^101^

A general mechanism of galvanic replacement for alloying process is as follow: when two dissimilar metals come into electrical contact in the presence of an electrolyte, corrosion of one of the metals is occurred. These metals form a galvanic couple, where one metal acts as the anode and the other as the cathode. The electrolyte facilitates the movement of ions, enabling the reaction. The metal with a lower electrochemical potential (more reactive) acts as the anode and undergoes oxidation (losing electrons). The metal with a higher electrochemical potential (less reactive) acts as the cathode and is protected from corrosion. The flow of electrons from the anode to the cathode drives the process. The reaction follows:M → M^*n*+^ + *n*e^−^where, M represents the anode metal, M^*n*+^ are its ions, and *n*e^−^ are the electrons released. The electrons released by the anode travel through the electrical connection to the cathode. At the cathode, reduction occurs as electrons combine with ions from the electrolyte, which is described by the following equation:O_2_ + 4H^+^ + 4e^−^ → 2H_2_O (in the presence of oxygen and acidic conditions).

Over time, the anode metal continues to lose material due to oxidation, leading to its degradation.

### 7.2. Chemical vapour deposition approaches

**Fig. 14:**
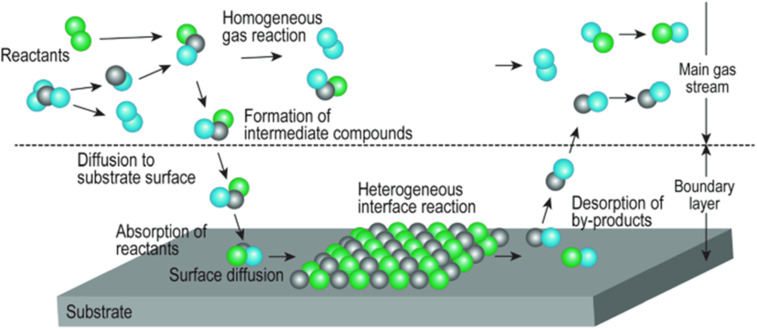
Schematic process of chemical vapour deposition for thin film preparation on the substrate's surface (adapted from ref. 109, © 2021 Springer, Singapore).

Chemical vapour deposition (CVD) is one of sophisticated method to prepare a thin film of material on the substrate from the molecular phase of the precursor. General process for CVD method for thin film growth on the substrate's surface is shown in Fig. 14.^109^ In the typical process, the vapour of reactants are transported into the reaction chamber, which is set to a certain temperature, using a gas carrier. In the chamber, the reaction is taken place amongst the reactant's vapour and produces an intermediate compound. The compounds will be adsorbed onto the substrate surface and undergoes heterogenous interfacial reaction to form thin film of the materials. By controlling the reaction condition, the substrate surface will be covered by the target material and by products will be desorbed from the surface. In the CVD process, the choice of precursor and deposition conditions such as temperature and gas flow rates significantly affect the quality and properties of the deposited films.^110,111^ Combinatorial techniques like metal–organic chemical vapor deposition (MOCVD) and pulsed spray evaporation CVD (PSE-CVD) are normally employed for depositing metals to obtain a smooth, continuous films with steady growth kinetics.^112^ These techniques can potentially be adapted for metal alloying on nickel film substrates.

Typical unique properties of CVD is laid its effectiveness for strain engineering. Controlled incorporation of alloying elements during vapor-phase growth induces lattice distortion, shifting the d-band center and optimizing orbital hybridization. This strain modulation directly lowers reaction barriers in HER and OER. CVD also enables controlled crystallinity, stabilizing metastable phases with enhanced conductivity and catalytic durability. Furthermore, the conformal nature of CVD minimizes unwanted oxide formation, offering surface passivation control that preserves active sites under harsh electrochemical conditions. Thus, CVD is not simply a deposition method but a mechanistic tool for band structure tuning and long-term stability.

However, CVD method to modify Ni substrate with metal, is presently limited. Nevertheless, some relevant details can be inferred from the general CVD processes of metals and nickel, which can be useful for understanding the deposition of metals on Ni. For noble metal film on Ni prepared using CVD, various precursors are used. For instance, for platinum (Pt), Pt(PF_3_)_4_ and (CH_3_C_5_H_4_)Pt(CH_3_)_3_ are normally used, while for Ni, Ni(CO)_4_ and Ni(CH_3_C_4_H_5_)_2_ or bis-(ethylcyclopentadienyl)nickel are used.^111,113^

### 7.3. Physical deposition methods

Physical deposition, particularly thermal or e-beam evaporation and sputter coatings are normally used for metal film growth on the substrate, including noble metals like gold (Au) and silver (Ag), onto various substrates.^114,115^ For the thermal of e-beam evaporation process, it involves heating the metal until it evaporates and then condenses onto the substrate, forming a thin film. The thin film quality can be controlled by proper substrate preparation, such as cleaning or polishing,^116^ and the deposition parameters, such as temperature and deposition rate.^115^ The thermal evaporation process can also produce nanoparticles and thin films with specific electrical properties.^114^ Post-deposition annealing is often applied to modify the structural properties of the films as it improves the crystallinity of the deposited layers.^117^ Optimizing the annealing temperatures and durations may enable the formation of desired film properties.^117,118^ Several important feature during the physical deposition of metallic thin film is presented in Table 2.

**Table 2:** Parameters that influences the film properties using physical deposition process

Key aspect	Details	Ref.
Thermal evaporation	Common method for depositing noble metals; involves heating and condensation	114 and 115
Substrate preparation	Polishing, etching, and cleaning are crucial; adhesion layers improve bonding	115 and 116
Structural characteristics	Morphology influenced by deposition parameters; nanoislands can be formed	115
Annealing	Affects structural properties; needs optimization to avoid degradation	117 and 118

It can be highlighted here that most of physical deposition techniques excel at defect density engineering. High-energy particle bombardment during the growth process introduces controlled dislocations and vacancies, which act as localized catalytic centers. Layered deposition also enables interface engineering, producing heterostructures with tailored charge transfer pathways and enhanced interfacial stability. Importantly, evaporation stabilizes uniform alloy distributions, suppressing segregation that often leads to deactivation. These mechanistic controls translate into improved electron transport, enhanced durability, and stabilized active sites for HER, OER, and CO_2_ reduction.

Regarding the sputtering process, there are two type of methods that is normally used for metal coating process. They include magnetron and reaction sputtering. Magnetron sputtering is frequently used for depositing metal and metal oxide films due to its advantages such as low substrate heating, high energy of sputtered atoms, and scalability for large surfaces. Meanwhile, reactive sputtering, it involves the introduction of reactive gases (*e.g.* oxygen, nitrogen) during the sputtering process to form compounds like nickel oxide or nickel nitride.^119–121^ The quality and properties of sputtered films are highly dependent on deposition parameters such as gas pressure, substrate temperature, and sputtering power.^122–124^ Achieving a uniform and defect-free morphology is crucial for the performance of sputtered films.

## 8. Conclusions and future perspectives

The field of electrocatalysis has witnessed significant advancements in recent years, particularly with the emergence of nickel-based alloys as promising candidates for enhancing the efficiency of crucial electrochemical reactions. Specifically, the hydrogen evolution reaction (HER) and oxygen evolution reaction (OER) are of paramount importance in energy conversion and storage applications, such as in fuel cells and electrolyzers.^125^ Nevertheless, to fully leverage the potential of these materials, several avenues for future research require exploration. Firstly, there exists a pressing need for further enhancement of both the intrinsic activity and structural characteristics of amorphous nickel-based alloys. Enhancing these properties is essential to improve their overall performance in facilitating HER and OER processes. The intrinsic activity pertains to the fundamental ability of the material to facilitate the desired electrochemical reactions, while geometry encompasses the structural attributes that influence the accessibility of active sites. Future studies should prioritize mechanisms that may yield substantial improvements in these dimensions, allowing for greater efficiency in energy generation and storage applications—an objective that is acutely relevant in the quest for sustainable energy solutions.

Secondly, a thorough examination of the roles played by various surface species in enhancing HER and OER activity and stability in amorphous nickel-based alloys is indispensable. Understanding these interactions can significantly contribute to the optimization of electrocatalytic performance. It is crucial to uncover how distinct surface species interact with the alloy's active sites and how these interactions influence reaction kinetics and overall stability. Such inquiries will pave the way for not only enhancing the performance of existing materials but also for formulating innovative strategies to develop novel compositions that could outperform current benchmarks. Additionally, developing efficient synthesis methods that can be scaled for industrial applications remains a critical challenge. Future research endeavors should thus focus on the establishment of robust and scalable synthesis techniques that maintain the high performance characteristics of these alloys, ensuring that advances in the laboratory can be translated into practical applications.

Finally, the design and creation of cost-effective and energy-efficient ultrathin two-dimensional (2D) bimetallic alloy nanosheets emerge as a promising frontier in the realm of electrocatalysis. Examining specific systems, such as nickel–molybdenum (Ni–Mo), nickel–germanium (Ni–Ge), and nickel–tin (Ni–Sn) bimetallic alloys, presents compelling opportunities for advanced research.^126^ The unique properties of 2D materials, such as high surface area and reduced material usage, make them particularly suited for electrocatalytic applications, allowing for enhanced performance with potentially reduced costs. Future investigations should aim to elucidate the specific attributes that these nanosheets bring to the table, including their catalytic performance, stability, and the mechanisms underlying their efficacy in HER and OER processes. Furthermore, extensive research into the synthesis and functionalization of these nanosheets will be vital in realizing their full potential as viable electrocatalysts.

One of the key challenges in designing Ni-based noble metal alloy catalysts lies in controlling both structure—such as morphology—and composition. Developing active sites using minimal amounts of noble and non-noble metals is a promising strategy but remains technically difficult.^127^ Pure Ni, while catalytically active, tends to promote the formation of graphitic carbon, leading to coking and catalyst deactivation.^128^ As a result, precisely engineering catalytic active sites through suitable preparation methods remains a major hurdle.^46^

An emerging approach involves incorporating multiple elements into single nanoscale alloys, such as high-entropy alloys (HEAs), which offer tunable catalytic properties through their complex composition.^129^ These heterogeneous HEAs represent a promising direction for discovering new catalysts across a range of reactions. Alloying Ni with small amounts of less reactive metals like Au has proven effective in suppressing coking and enhancing catalyst longevity.^128^ Similarly, the single-atom alloy approach—such as dispersing isolated Ni atoms in a Cu matrix—has shown great potential for improving catalytic selectivity and resistance to poisoning.^128^

Non-noble trimetallic alloys also show promise. For example, Ni_3_Ga_0_._8_In_0_._2_/SiO_2_ catalysts significantly boost reaction rates, demonstrating how careful compositional tuning can enhance performance.^46^ Likewise, NiRu alloys anchored on nitrogen-doped carbon-coated TiO_2_ have shown exceptional activity in ammonia borane (NH_3_BH_3_) hydrolysis, underscoring the advantages of Ni-noble metal systems in catalysis.^127^ Despite these advancements, challenges persist—particularly in developing synthesis methods that enable precise control over active site formation, maximize atomic utilization, and improve reaction efficiency.^46,127^

## Author contributions

Akrajas Ali Umar: design the concept of the review; writing – draft; finalizing the revision and submission. Munetaka Oyama: supervise and reviewing the manuscript.

## Conflicts of interest

The authors declare no conflict of interest.

## Data Availability

No primary research results, software or code have been included and no new data were generated or analysed as part of this review.
